# PSMA PET/CT guided intensification of therapy in patients at risk of advanced prostate cancer (PATRON): a pragmatic phase III randomized controlled trial

**DOI:** 10.1186/s12885-022-09283-z

**Published:** 2022-03-08

**Authors:** Cynthia Ménard, Sympascho Young, Katherine Zukotynski, Robert J. Hamilton, François Bénard, Steven Yip, Christopher McCabe, Fred Saad, Michael Brundage, Roy Nitulescu, Glenn Bauman

**Affiliations:** 1 Department of Radiation Oncology, Centre Hospitalier de l’Université de Montréal (CHUM), Montreal, Quebec Canada; 2 Department of Oncology, London Health Sciences Centre, Western University, London, ON Canada; 3 Departments of Medicine and Radiology, McMaster University, Hamilton, ON Canada; 4 Department of Medical Imaging, Western University, London, ON Canada; 5 Department of Radiology, University of British Columbia, Vancouver, BC Canada; 6 Department of Surgery (Urology), Princess Margaret Cancer Centre, University of Toronto, Toronto, ON Canada; 7 Department of Radiology, British Columbia Cancer Agency, University of British Columbia, Vancouver, BC Canada; 8 Department of Oncology, Tom Baker Cancer Centre, University of Calgary, Calgary, AB Canada; 9 Institute for Health Economics, University of Alberta, Edmonton, AB Canada; 10 Department of Oncology, Kingston Regional Cancer Centre, Queen’s University, Kingston, ON Canada; 11 CHUM Center for the Integration and Analysis of Medical Data (CITADEL), Quebec, Canada

**Keywords:** PSMA PET, Prostate cancer, Phase III clinical trial, Cost-effectiveness analysis, Failure free survival, Overall survival, Radiotherapy, Superiority

## Abstract

**Background:**

Positron emission tomography targeting the prostate specific membrane antigen (PSMA PET/CT) has demonstrated unparalleled performance as a staging examination for prostate cancer resulting in substantial changes in management. However, the impact of altered management on patient outcomes is largely unknown. This study aims to assess the impact of intensified radiotherapy or surgery guided by PSMA PET/CT in patients at risk of advanced prostate cancer.

**Methods:**

This pan-Canadian phase III randomized controlled trial will enroll 776 men with either untreated high risk prostate cancer (CAPRA score 6–10 or stage cN1) or biochemically recurrent prostate cancer post radical prostatectomy (PSA > 0.1 ng/mL). Patients will be randomized 1:1 to either receive conventional imaging or conventional plus PSMA PET imaging, with intensification of radiotherapy or surgery to newly identified disease sites. The primary endpoint is failure free survival at 5 years. Secondary endpoints include rates of adverse events, time to next-line therapy, as well as impact on health-related quality of life and cost effectiveness as measured by incremental cost per Quality Adjusted Life Years gained.

**Discussion:**

This study will help create level 1 evidence needed to demonstrate whether or not intensification of radiotherapy or surgery based on PSMA PET findings improves outcomes of patients at risk of advanced prostate cancer in a manner that is cost-effective.

**Trial registration:**

This trial was prospectively registered in ClinicalTrials.gov as NCT04557501 on September 21, 2020.

**Supplementary Information:**

The online version contains supplementary material available at 10.1186/s12885-022-09283-z.

## Background

Prostate cancer (PCa) is the most common non-skin malignancy and the fifth leading cause of cancer death in men worldwide [1]. High-risk PCa at diagnosis and biochemically recurrent disease are both associated with poor rates of failure-free survival (FFS) with current treatment strategies [2–4]. We postulate that the low FFS is, at least in part, due to occult metastases in men undergoing curative therapy for localized disease. Until recently, conventional imaging (CI) for staging of PCa has consisted of a bone scan and abdominal-pelvic computed tomography (CT). However, conventional imaging has known limitations in sensitivity and specificity for PCa. In the last decade, “Next Generation Imaging” tools have been developed [5]. Of particular interest, prostate specific membrane antigen targeted positron emission tomography (PSMA PET) has emerged as a promising technique for imaging PCa. It uses radiopharmaceuticals that target PSMA, a type II transmembrane glycoprotein that is overexpressed in PCa cells compared to other tissues. PSMA expression correlates with a number of important metrics of PCa tumor aggressiveness including Gleason score, propensity to metastasize and the development of castration resistance. PSMA-directed PET, either combined with CT (PET/CT) or magnetic resonance imaging (PET/MRI), is thought to be sensitive and specific for the detection of metastatic PCa and is beginning to make its way into clinical practice worldwide.

The current literature suggests PSMA-directed PET/CT has superior sensitivity and specificity compared to CI. A meta-analysis of 37, mostly retrospective, studies including 4790 patients found the sensitivity and specificity of PSMA PET was 75 and 99% respectively in the context of primary staging [6]. A prospective crossover trial found 27% greater accuracy for PSMA PET compared to CI, suggesting PSMA PET was more accurate for detecting pelvic lymph node and distant bone metastasis [7]. Another prospective trial, with histopathologic correlation, found a 92% sensitivity per patient in the context of restaging for biochemical failure (BF) [8]. One of the benefits of PSMA PET is its apparent high sensitivity at low PSA, with detection rates of 75% when PSA is in the range of 1.00–1.99 ng/mL and 59% when PSA is in the range of 0.5–0.99 ng/mL [6]. CI, on the other hand, does not typically detect lesions at PSA levels < 10 ng/mL [9, 10].

More recently, several studies have explored the effect of PSMA PET on clinical management [11]. A prospective multicentre study of 431 men found that the additional information from PSMA PET changed management plans in 51% of patients (21% in patients undergoing primary staging and 62% of those with BF) [12]. A prospective study investigating the use of PSMA PET/CT prior to radical prostatectomy (RP) in 103 high-risk patients showed a change in surgical management in 12.6% of men [13].

Although there is robust evidence that PSMA PET/CT offers improved disease detection compared to CI, there is a paucity of literature on whether PET-guided management ultimately translates into improvement in cancer control and survival outcomes. Given its improved accuracy in detecting early metastases, we hypothesize PSMA PET guided intensification of therapy will yield improved FFS in primary and recurrent PCa [14]. In addition, by identifying the minority of men with more extensive metastatic disease, early systemic therapy and/or metastasis directed therapy can be deployed, thus potentially improving FFS and quality of life (QoL) by avoiding futile local therapy and its associated side effects. In May 2018, we launched a prospective randomized phase II trial PSMA-PET guided intensification of radiotherapy studying the potential impact of PSMA PET/CT guided radiotherapy on outcomes of men deemed at high risk of harbouring undetected metastatic disease (NCT03525288). That study has completed accrual of 253 patients and preliminary results showed approximately half of patients who had PSMA PET/CT had new lesions detected that led to an intensification of radiotherapy, with no difference in the rate of grade 2 or more early toxicity outcome [15].

In this next-step prospective multi-centre pan-Canadian randomized phase III trial, we will test the hypothesis that intensification of RT or surgery based on the result of PSMA-PET will improve cancer control outcomes. We will also test the hypothesis that it is a cost-effective intervention, with minimal toxicity and overall improvement in quality of life in the long-term. This trial builds upon our collective experience in several different clinical trials, where our team of investigators has evaluated PSMA PET/CT in Canadian patient populations [16–19].

## Methods/design

This study is centrally approved by the Quebec Multicentric Ethics Committee at the sponsor site (CHUM – approval number MP-02-2021-9258). The trial is funded by the Canadian Cancer Society with in-kind radiotracer provided in part by Lantheus Medical.

### Objective

To determine if PSMA PET/CT guided intensification of therapy is superior to therapy based on conventional imaging as measured by improved failure free survival and other cancer control outcomes, and whether it is associated with differences in rates of toxicity or quality of life.

### Study design

**Fig. 1:**
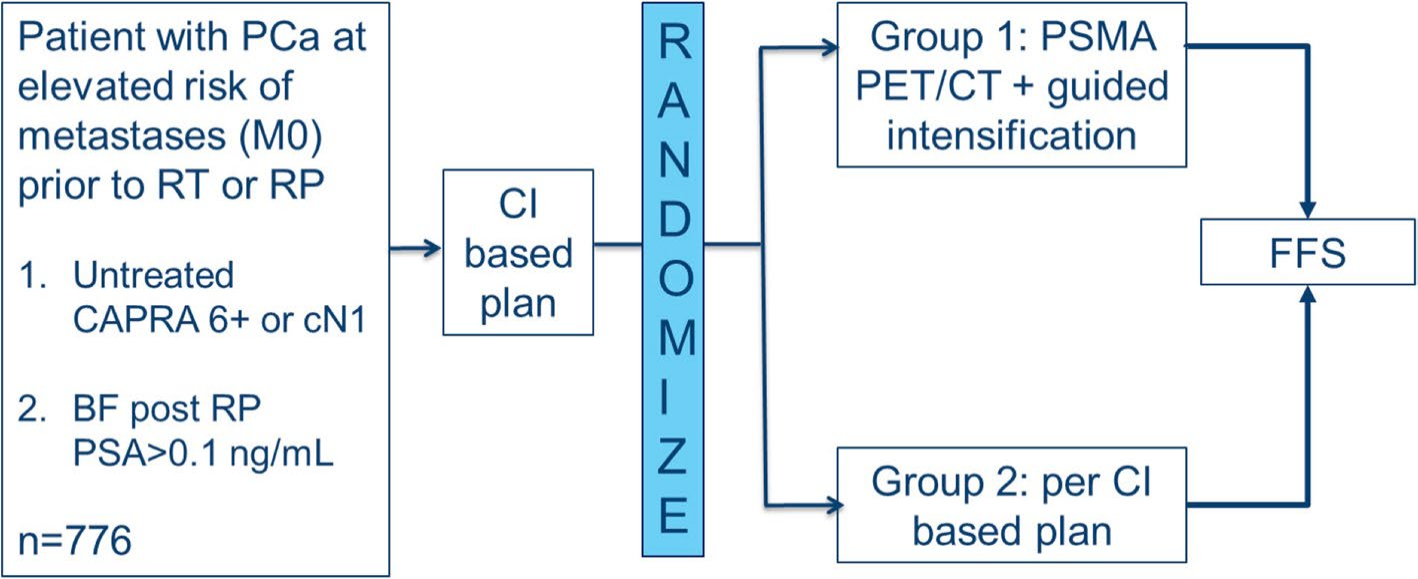
General study schema. (CI = conventional imaging)

This is a prospective randomized, multi-centre, phase III superiority trial comparing PSMA PET/CT guided intensification of therapy vs. conventional imaging guided therapy in patients with high risk PCa, on the basis of UCSF’s Cancer of the Prostate Risk Assessment (CAPRA) score [20] or clinical node positive by conventional staging, who are being offered primary RT or RP or men with BF post RP who are being offered salvage RT (Fig. 1).

Patient randomization will be stratified according to:

1. Therapeutic cohort (high-risk RT, high-risk RP, salvage RT post RP)
2. Planned use of pelvic lymph node radiotherapy (PLNRT) among patients receiving RT
3. Planned use of hormone therapy (HT) in the patient’s conventional imaging guided treatment plan.

Patients will be randomized 1:1 to receive PSMA PET/CT by variable block randomization through CASTOR’s Electronic Data Capture (EDC) system. Participants will be enrolled by study personnel at each participating institution and assigned to study arm per randomization. This is an intent-to-treat analysis and all randomized at-risk patients will be included in the primary analysis. A significance level of 0.05 will be used to determine significance in all analyses.

#### Study endpoints

Primary endpoint:

- Failure-free survival, defined as time to BF, local/regional failure (pathology or conventional imaging), distant metastasis (conventional imaging), or death from any cause.
  - ° Biochemical failure (which is expected to constitute the majority of the events) is defined as follows:
    - ▪ High-risk Post RT: Phoenix definition (PSA > PSA nadir + 2 ng/mL)
    - ▪ High-risk Post RP: PSA ≥0.1 ng/mL
    - ▪ Salvage RT post RP: PSA nadir + 0.2 ng/mL

Secondary endpoints:

- Adverse events (AE), as measured by the Common Terminology Criteria for Adverse Events (CTCAE) v.5
- Biochemical failure (BF)
- Local failure or regional failure on conventional imaging
- Distant metastasis on conventional imaging
- Prostate Cancer Specific Survival
- Overall Survival (OS)
- Time to subsequent next-line therapy
- Health-related quality of life (HRQoL), as measured by Expanded Prostate Cancer Index Composite 26 (EPIC-26) and EORTC Core Quality of Life Questionnaire (QLQ-C30)
- Detection yield: proportion of patients where PSMA PET/CT identifies a previously undetected lesion.
- Rate and nature of treatment intensification.
- Cost effectiveness: incremental cost per Quality-Adjusted Life Years (QALY) gained

#### Patient selection

##### Inclusion criteria

- Histological diagnosis of adenocarcinoma of the prostate planned for curative-intent standard-of-care RT (primary or salvage post RP) or RP with lymph node dissection.
- Age ≥ 18
- High risk of regional or distant metastases as defined by any of:
  - ° Newly diagnosed and untreated PCa with CAPRA score 6–10, or stage cN1.
  - ° Prior history of RP and BF (PSA > 0.1 ng/mL).
- Patients must provide study-specific informed consent prior to study entry.
- Eastern Cooperative Oncology Group (ECOG) performance status ≤2

##### Exclusion criteria

- Active or prior androgen deprivation therapy (except 5-alpha reductase inhibitor) terminated < 12 months prior to enrollment.
- Prior or planned PSMA PET/CT scan outside of this clinical trial.
- Charlson Comorbidity Index > 5 (see Appendix 2).
- Prior curative intent treatment for PCa with local therapy other than surgery (primary RT or ablative therapies)
- Evidence of extra-pelvic nodal disease (M1a) on conventional imaging (if performed)
- Evidence of metastatic disease (M1b bone, M1c viscera/soft tissue) on conventional imaging (if performed)

#### Pre-treatment evaluation

Within 120 days prior to randomization:

- History and targeted physical examination (as needed), including prior cancer therapies
- Charlson comorbidity index
- Performance status
- Conventional imaging guided treatment plan documented
- PSA (within 60 days)

#### Randomization

Prior to treatment (RT or surgery)

- EPIC-26, QLQ-C30, and EQ-5D-5L questionnaires
- Adverse event evaluation, as measured by the CTCAE v.5
- Concomitant medications assessment
- Imaging and pathology reports uploaded to Electronic Data Capture system
- Tissue and blood biobanking (if available at institution)

#### Evaluation during treatment

- Adverse event evaluation, as measured by the CTCAE v.5

#### Follow up

Follow-up is planned at months 3 and 6, then annually after treatment. The following investigations should be performed:

- Adverse event evaluation and concomitant medications assessment with each follow up
- PSA at each follow-up
- EPIC-26, QLQ-C30, and EQ-5D-5L questionnaires at months 3 and 6, and years 1, 2 and 5 post-treatment completion
- Digital rectal exam (DRE), follow-up imaging and biopsies as clinically indicated

#### Intervention

##### General information

Patients allocated to the experimental study arm will undergo either a PSMA PET/CT or PET/MRI prior to treatment. Treatment (RT or RP) should be initiated within 10 weeks of randomization.

##### Conventional imaging guided intervention

A conventional imaging guided treatment plan will be defined for all patients prior to randomization. Those patients randomized to the conventional imaging arm will have their treatment planning and delivery based on conventional imaging (i.e. non-PSMA PET/CT based) as per investigator discretion. For high risk patients planned for RP, pelvic lymph node dissection (PLND) is recommended. For patients receiving primary or salvage RT, use of regional pelvic nodal radiation and adjuvant HT is at the investigator’s discretion and will be documented on a per patient basis.

### PSMA PET/CT based intervention

#### PET/CT imaging

##### Investigational PET imaging radiotracer

This study will use 18F radiopharmaceuticals (18F-DCFPyL or 18F PSMA 1007) as available to participating sites through existing Health Canada approved supply arrangements. 18F labelled radiopharmaceuticals possess several advantages over 68-Gallium tracers: lower energy and shorter positron range allowing for higher image resolution, longer half-life (110 min vs. 68 min), the possibility of generating multiple doses from a single cyclotron synthesis run, and easier shipping to sites without radiochemistry/cyclotron facilities.

##### PET/CT imaging protocol

Imaging is to be performed on the institution’s most modern scanner, preferably with time-of-flight capabilities, within 3 weeks of randomization. The radiopharmaceutical will be injected by slow intravenous (IV) push followed by saline flush. Weight based (2–4 MBq/kg) and standardized dose (300 ± 60 MBq) approaches are allowed as per local practice. Images will be acquired 60–120 min following tracer injection. CT and PET images from the base of the skull to the proximal thighs will be acquired sequentially. Repeat imaging of equivocal sites of disease can be obtained up to 180 min post-IV.

Low-dose, non-contrast enhanced CT will be acquired along with the PET imaging, and will be used for attenuation correction and anatomical localization. For the PET, the number of beds and time-per-bed will vary according to the patient’s height, weight and scanner recommendations. Exact parameters should conform to the local standard-of-care, but typical parameters in the average patient are 6–8 bed positions, and approximately 3 min per bed position. Administration of IV furosemide (10–40 mg) prior to imaging or prior to repeat imaging to help clear bladder and ureteral activity is optional.

##### PET/CT interpretation, reporting and review

Images will be interpreted locally by an experienced nuclear medicine specialist with full knowledge of clinical history, and prior imaging. In addition to a standard local clinical report, an electronic Case Report Form (eCRF) will be completed where sites of suspected disease on PET will be correlated with CT findings on the PET/CT and a 4-point scale will be used to establish a standardized metric for the probability of malignancy, specifically: 1 = benign, 2 = equivocal, 3 = probably malignant, 4 = definitely malignant. All cases will be independently reviewed centrally for the purpose of quality control prior to any interventions.

### PSMA PET/CT based intensification

**Fig. 2:**
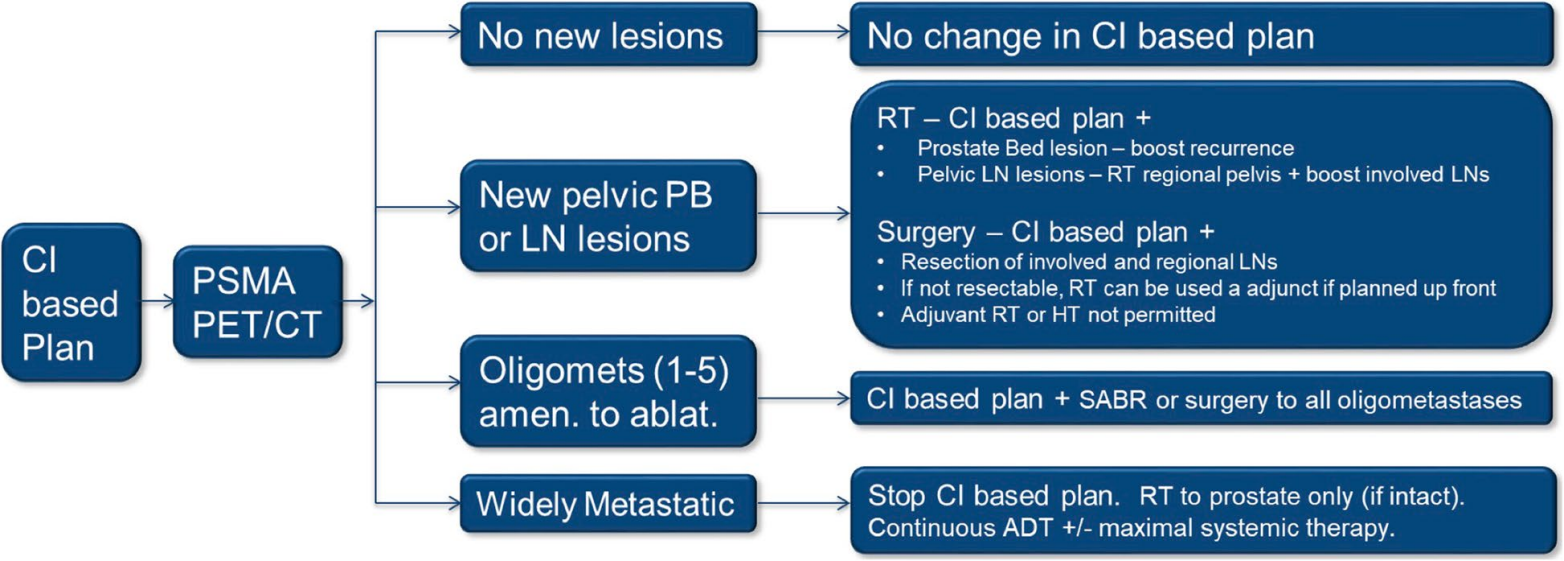
Schema for intensification of therapy by PSMA PET findings (CI = conventional imaging)

Following local and central reads, results of the PSMA PET will be provided to the treating physician and the treatment plan intensified as appropriate to the PSMA PET findings as outlined in Fig. 2 and below. Note, in no case will de-intensification from the pre-PSMA PET pre-specified conventional imaging based plan be allowed. For example, for a patient who is planned for RP with PLND, RP without PLND is not allowed in the case of a negative PET. Similarly if regional nodal radiotherapy or adjuvant HT was part of the conventional imaging guided plan, those elements would continue even if the PET demonstrated no extra-prostatic disease.

#### No new lesions detected on PSMA PET

If no new lesions are detected, RT or surgery to proceed as planned based on conventional imaging. De-intensification of therapy is not permitted.

#### New local/regional lesions detected on PSMA PET

All visible lesions (probably and definitely malignant) to be included in a definitive treatment plan. Intraprostatic lesions are not considered new lesions unless they extend into seminal vesicles in a manner previously unknown.

##### Surgery

Proceed with RP, resection of involved lymph nodes, and regional lymph node dissection. If involved lymph nodes are not resectable due to surgical constraints, RT to involved LNs (including elective dose to the involved chain) is permitted but must be planned up front as part of first-line therapy in combination with surgery. Note that adjuvant RT in the absence of BF is not permitted.

##### Radiotherapy

Proceed with planned prostate/prostate bed (PB) RT +/− pelvic nodal RT as planned. (see Guidelines for Intensified Radiotherapy below).

#### Oligometastases (1–5 sites on PSMA PET) amenable to ablative therapy

Proceed with conventional imaging based radiotherapy or surgery. In addition, all detected metastatic lesions to be treated with SABR or surgery. For the purposes of this study, PET identified lesions that are contiguous (i.e. adjacent nodes in the same nodal chain; adjacent vertebral body lesions) will constitute 1 site.

#### Widespread metastatic disease not amenable to ablation

Treatment (RT) of primary disease (if present) is recommended. A change to continuous HT is also recommended, allowing local investigators discretion regarding additional Health Canada approved systemic therapy (e.g. abiraterone acetate, apalutamide, enzalutamide, docetaxel) after discussion with lead study PI’s. Ablative therapy to metastatic sites is not permitted, but palliative RT for symptom management is permitted.

##### Guidelines for intensified radiotherapy

Intensification of RT dose for detected lesions: Recommended dose objectives in for intensification of detected lesions on PSMA PET/CT are described in the following table in terms of equivalent dose in 2Gy per day fractions (EQ2D) based on the Linear Quadratic model of cancer cell survival and assuming an alpha/beta of 1.4. Specific techniques (for example inclusion of a brachytherapy boost) and fractionation (for example ultra-hypofractionated stereotactic RT) are at the discretion of the treating radiation oncologist in keeping with institutional standard of care practice unless otherwise specified. All institutions are required to undergoing credentialing with a standardized salvage RT case with dose intensification to a PSMA PET/CT involved lymph nodes. Participating centres are also required to have been previously credentialed for SABR for metastasis directed therapy through a prior REB approved clinical trial. Centres without local PSMA PET/CT or SABR capability are permitted to partner with other participating institutions for delivery of that component of care where needed. Given the safety and efficacy of dose escalation to imaging defined dominant intra-prostatic lesions as noted in phase II/III trials of mpMRI based DIL delineation [21, 22]; techniques that utilize a concomitant boost of DIL to allow escalation beyond EQ2D 78Gy to the whole gland are strongly recommended.

**Table:** 

Target	Target Dose Range (Gy EQD2)	Fractionated RT (Gy EQD2)	HDR/LDR Brachytherapy Boost (Gy EQD2)	SABR (Gy EQD2)
**Prostate tumor** **< 50% of prostate volume**	(78–168)	(78–113) e.g. 77Gy/35 fractions with concomitant boost to 95Gy/35 fractions [21]	(115–140) intraprostatic boost optional at time of brachytherapy, (125% - GTV)	(92–168) e.g. 35Gy/5 fractions with concomitant boost to 50Gy/5 fractions [22]
**Involved pelvic or adjacent PA LNs (up to T12/L1)**	(60–86)	(60–74) concomitant integrated or sequential boost (aim as high as possible within dose range while respecting OARs)	na	(65–86) 30–35/5# Concomitant boost
**Prostate bed GTV**	(70–74)	(70–74) sequential boost only	na	na
**Bone metastasis**	(66–110)	(66–74) concomitant if adjacent to pelvic RT fields	na	(66–110) 30-40Gy/5# 24Gy/2# (spine only)
**Remote LNs**	(66–110)	na	na	(66–110) 30-40Gy/5#
**Lung mets (peripheral)**	(190)	na	na	(190) 48Gy/4#
**Lung mets (central)**	(86–168)	na	na	(86–168) 35-50Gy/5#
**Liver metastasis**	(66 or more)	na	na	(66 or more) 30-60Gy/3–5#

For gross disease that has achieved a complete radiographic response to hormone therapy (e.g. enlarged lymph nodes), the original area of the disease should be treated to an EQD2 of 42-56Gy

In general for stereotactic radiotherapy of metastases, a dose of 35 Gy in 5 fractions delivered in a conformal fashion and with dose homogeneity is an effective and safe dose for the majority of non-spine tumour targets. For spine metastases, a dose of 24 Gy in 2 fractions is highly recommended

Radiation therapy details including OAR constraints are available in Appendix

*See below for OAR constraints included in Appendix:*

*Organs at Risk*

Major – Considered a planning priority (compromise PTV coverage to respect)

Minor - Strive to achieve WITHOUT compromising PTV coverage

In general, for OARS situated well below deviation thresholds, please apply the principle of As Low As Reasonably Achievable (ALARA) without compromising PTV coverage.

*Sequential sum plans (final combined plan)*

**Table:** 

(Gy)	Rx 1.8-2Gy/Day		Rx 2.5Gy/Day		Rx 2.7Gy/Day		Rx 3Gy/Day	
	Major	Minor	Major	Minor	Major	Minor	Major	Minor
**Rectum**								
D50%	47	43	43	39	42	38	40	36
D25%	66	60	60	55	58	53	54	49
D15%	74	67	67	61	65	59	58	55
D0.5cc	78	76	71	69	68	66	60	59
**Bladder**								
D50%	50(65)	45(55)	46(60)	42(50)	45	41	42	38
D25%	66(68)	61(64)	60(65)	55(60)	58	54	55	50
D15%	76	70	69	63	66	61	60	57
**Bowel_bag**								
D200cc	48	44	44	41	43	40	40	37
D3cc	56	50	51	46	50	45	47	42
D0.1cc	66	58	60	53	58	51	54	48
**Femoral Heads**								
D1%	55	40	50	37	49	36	46	34

The values in bracket are for RT salvage post-prostatectomy with PB targets

*Single plans (concomitant)*

**Table:** 

**(Gy)**	5 fractions		15 fractions		20 fractions		22 fractions	
	Major	Minor	Major	Minor	Major	Minor	Major	Minor
**Rectum**								
D50%	25	23	37	33	40	36	41	37
D25%	31	29	50	45	54	49	56	51
D15%	34	32	55	50	58	55	63	57
D0.5cc	36	35	57	56	60	59	65	64
**Bladder**								
D50%	27	24	39	35	42	38	43	39
D25%	33	30	50	46	55	50	57	52
D15%	37	34	56	52	60	57	64	59
**Bowel_bag**								
D200cc	25	24	37	34	40	37	42	38
D3cc	29	26	43	38	47	42	48	43
D0.1cc	33	29	49	44	54	48	56	50
**Femoral Heads**								
D1%	28	22	42	32	46	34	47	35
**Gy (post RP)**	25 fractions		28 fractions		33 fractions		39 fractions	
	Major	Minor	Major	Minor	Major	Minor	Major	Minor
**Rectum**								
D50%	42	38	43	39	45	41	47	43
D25%	58	53	60	55	63	57	66	60
D15%	65	59	67	61	71	64	74	67
D0.5cc	68	66	71	69	74	72	78	76
**Bladder**								
D50%	45 (60)	41 (50)	46	42	48 (65)	44 (55)	50	45
D25%	58 (65)	54 (60)	60	55	63 (68)	58 (64)	66	61
D15%	66	61	69	63	72	66	76	70
**Bowel_bag**								
D200cc	43	40	44	41	46	42	48	44
D3cc	50	45	51	46	54	48	56	50
D0.1cc	58	51	60	53	63	56	66	58
**Femoral Heads**								
D1%	49	36	50	37	53	39	55	40

The values in bracket are for RT salvage post-prostatectomy with PB targets

### Statistics and sample size calculations

#### Primary endpoint

Failure free survival (FFS) is defined as time from date of randomization to the date of BF, local/regional failure (pathology or conventional imaging), distant metastasis (conventional imaging), or death from any cause, whichever occurs first, or last known follow-up date. Event-free patients are censored at their last known follow-up date.

#### Primary endpoint analysis plan

FFS will be estimated using the Kaplan-Meier method and treatment arms compared using the stratified log-rank test. Stratification will be performed, for all analyses, using the following therapeutic cohort definitions: 1) high-risk RT, 2) high-risk RP, 3) salvage RT post RP without PLNRT, and 4) salvage RT post RP with PLNRT. A stratified Cox proportional-hazards model will be used to determine the hazard ratio (i.e., treated vs. controls) and to assess the effects of stratification factors [23]. The primary endpoint will be reported after the first of the following occurs: (1) it has been 5 years since the end of the accrual period of the study, or (2) there have been a total of 213 failure events reported (i.e., the expected number of events as per our sample size calculation reported below).

#### Secondary endpoints and analysis plans

##### Cancer control/efficacy

Overall survival will be estimated using the Kaplan-Meier method and treatment arms compared using the stratified log-rank test. The cumulative incidence estimator will be used to estimate time to event distributions for endpoints with competing risks (BF, local and regional failure, distant metastasis on conventional imaging, and cause-specific mortality). For all efficacy endpoints, Cox proportional hazards models will be used to determine hazard ratios (cause-specific hazard ratios in the case of endpoints with competing risks) and to assess the effects of stratification factors and other covariates of interest. All efficacy endpoints will be reported once 213 FFS events have occurred.

##### Adverse events

AEs will be graded using CTCAE v5. The number of patients with at least 1 grade 3 or higher AE will be compared between the treatment arms. All comparisons will be tested using a Chi-Square test.

##### Quality of life

Patients’ quality of life (QoL) will be assessed using the validated EPIC-26 and QLQ-C30 questionnaires. The QoL data will test the null hypothesis that guided treatment intensification results in no statistically significant differences between intervention arms for the four domain scores of the EPIC-26 instrument nor for the functional scale scores of the QLQ-C30. Any statistically significant differences will be interpreted for clinical significance by comparing them to the reported minimally important clinical differences for each instrument’s main domains.

##### Cost-effectiveness analysis

The primary analysis of economic evaluation will adopt the 5-year time horizon of the trial and will compare direct health care system costs incurred by patients in the two arms of the trial, taking the perspective of the Canadian Public Heath Care System. The primary effectiveness outcome for this analysis will be FFS at 5 years (5YFFS). We will calculate the expected incremental cost per 5YFFS. A number of secondary analyses will be conducted including (a) using QALYs as the outcome measure; (b) out-of-pocket and productivity costs incurred by patients as well as health system costs; and (c) caregiver/household spillover effects in the measures of both cost and outcome.

### Statistical assumptions and sample size calculations

Participants are expected to be distributed among the four therapeutic cohorts in the following proportions: High-Risk RT, 40%; High-Risk RP, 25%; salvage RT (PB only) post RP, 15%; and salvage RT (PB + PLN) post RP, 20%. For each cohort, conventional imaging arm 5YFFS estimates are 80% for high risk RT [3, 4, 24, 25], 40% for high risk RP [26], 75% for salvage RT to PB alone [2], and 85% for salvage RT to PB with nodal radiation [2], respectively, for a pooled average of 70%.

Furthermore, we conservatively estimate the new lesion detection rate in the pooled PSMA PET/CT cohort to be 40% (per NCT03525288). Of these, 50% consist of lesions that would not have been treated with curative therapy, and would be amenable for definitive intensified therapy. Therefore at least 20% of patients in the experimental arm would potentially derive direct benefit from therapy intensification (untreated vs. treated). If we conservatively assume treatment of these disease sites to be between 40 and 45% effective in improving 5YFFS outcomes, we would expect to observe an absolute risk reduction between 8 and 9% in FFS, corresponding to a hazard ratio between 0.66 and 0.70, approximately.

In terms of modeling assumptions, we used a stratified, exponential, proportional-hazards model. Accrual was assumed to occur uniformly over 30 months following a 6- month ramp-up period of negligible recruitment. Following the accrual phase, the follow-up phase will last 5 years. Attrition was assumed to be 10% over the 5 years of follow-up. Our target was 80% power with a two-sided alpha of 5%. We estimate a total sample size requirement of 776 subjects (213 outcome events) in order to detect a hazard ratio of 0.68 (28% relative risk reduction and 8.4% absolute risk reduction in 5YFFS) over the pooled study sample and maintain adequate power under our assumptions. The 5YFFS is expected to be 21% in the experimental PSMA PET/CT arm, translating into a 79% failure-free rate at 5 years in the experimental arm vs. 70% in the conventional imaging arm.

### Accrual/study duration considerations

This study is expected to accrue 30 patients per month so accrual would be completed in 30 months, after a 6-month ramp up period of negligible accrual. Full information for the FFS endpoint is expected to be mature for analysis approximately 6 years from study activation. Our recruitment plan includes pre-specified modifications if early accrual suggests that we are likely to fall short of our recruitment target. Accrual will be evaluated by the Data and Safety Monitoring Committee (DSMC) every 6 months following ramp-up. Interim accrual figures will be used to project accrual into the future. If our projections suggest that it is likely we will reach our accrual target within 3 years of study activation, the trial will continue without modifications. However, if our projections suggest it is likely we will reach our accrual target between 3 and 5 years from study activation, we will apply mitigation strategies as determined by the study executive. Finally, if our projections suggest we are unlikely to reach our accrual target within 5 years of study activation, we will consider potentially stopping the trial early.

### Data and safety monitoring committee

The DSMC will meet twice a year at a minimum after study initiation to review patient accrual, general data quality, study conduct, and morbidity/adverse events. The DSMC will review reports of any serious adverse events and relevant charts to determine if such toxicity is related to treatment. If the DSMC deems that toxicity rates are excessive, then the DSMC can make a recommendation to modify, suspend or terminate the study. Membership of the DSMC includes representation from radiation oncology, medical oncology, surgical oncology, biostatistics, clinical research staff and ex-officio. Any members with potential conflicts of interest must withdraw from decision-making discussions related to the study.

### Access to data

The final dataset will be available for public access. There are no contractual agreements limiting any investigator access to trial data.

## Discussion

This phase III pragmatic multi-institutional randomized trial seeks to investigate the impact of PSMA PET guided intensification of surgery or radiotherapy on cancer control outcomes in men with high risk untreated PCa and BF post RP. We believe that in spite of promising data regarding superior diagnostic performance of PSMA PET and its influence on clinical management, ultimately, robust data on patient outcomes when curative-intent therapies are intensified by PSMA PET results are necessary considering the significant upfront costs and potential risk of intensification [27]. In addition, we seek to measure other impacts of PSMA PET-intensification of therapy, including rates of toxicity, quality of life, and cost-effectiveness as measured by incremental cost per QALY gained.

We believe that one of the reasons high-risk and recurrent PCa is associated with poor rates of FFS is due to our inability to accurately diagnose and localize early metastatic spread in these patients using conventional imaging. If patients with early metastatic disease can be accurately diagnosed and treated accordingly, these patients may receive timely treatment before further spread of disease and also be spared the toxicities of next-line therapy. Our hypothesis is supported by the recent results of a randomised controlled trial EMPIRE-1, which indicated that 18-F-fluciclovine-PET guided radiotherapy may improve event-free survival at 3 years for patients with BF post RP, without increasing toxicity [14]. However, results were not analysed in intent-to-treat, and PSMA PET is favoured to be superior to 18-F-fluciclovine, especially at low PSA levels when patients are more likely to have localized and potentially curative disease [28, 29].

In designing this trial, we chose FFS as the primary outcome measured, with other measures of survival as secondary outcomes. There are a number of reasons for using this surrogate marker as the primary outcome. First, pragmatically it allows for detecting a statistically significant difference using a reasonable number of patients and relatively shorter length of follow-up, as our goal is to obtain the evidence in order to aid governments and health authorities considering investing in PSMA PET in a timely manner. Second, FFS is a clinically relevant endpoint as it affects patients by causing psychological distress and usually triggers the next line of therapy, whether salvage RT with local urinary and bowel side effects or androgen-deprivation therapy with side effects of fatigue, hot flashes and sexual dysfunction. We acknowledge that other measures of survival, such as metastasis-free survival, may be more closely correlated with overall survival, and will be measured as secondary outcomes [30].

Our study population was also carefully considered. We included patients considered for curative-intent treatment at high risk for regional or distant metastases, whether in the primary disease or BF setting. Instead of using Gleason Score or Grade Group as the sole determinant of risk of metastasis in the primary setting, we used the CAPRA score, which is a nomogram validated to predict metastases, PCa-specific mortality and all-cause mortality [31]. For patients with BF post RP, we used a low cut-off of PSA > 0.1 ng/mL instead of the more common definition of ≥0.2 ng/mL because it consists of an actionable threshold for next-line salvage intervention. In addition, those with poor performance status (ECOG > 2) are excluded, as these patients are typically not suitable for curative intent treatment and were more likely to have significant comorbidities that may affect survival rates.

Finally we must emphasize that the two radiotherapy study arms of this trial are identical to our prior Phase II trial (NCT03525288). In this regard, a planned meta-analysis of the phase II and III trials will substantially increase our power to detect a difference attributable to the intensification of radiotherapy.

Recently, other phase II-III randomized trials have also been launched to investigate similar questions about the effect of PSMA PET-guided therapy on cancer control outcomes [14]. There are two UCLA trials PSMA-dRT (NCT04457245) and PSMA SRT (NCT03582774) studying primary staging and salvage radiotherapy guided by PSMA PET respectively, Swedish trial NCT04794777 trial studying therapy in the BF setting, and the U.S. Veteran’s VA STARPORT trial NCT04787744 focusing on the oligo-recurrent setting. The results of PATRON will add to this developing evidence base to best understand where PSMA PET has its largest impact on clinical outcomes.

## Conclusion

PATRON is a pan-Canadian phase III pragmatic multi-institutional randomized trial leveraging the ability of PSMA PET to better detect early sites of metastases in patients with high risk and biochemically recurrent PCa in the real world setting. The aim of the trial is to investigate whether treatment intensification guided by such imaging would improve cancer control outcomes, quality of life, and reduce toxicity in a cost-effective manner. Such results provide the required evidence as health authorities consider a shift from conventional PCa imaging to PSMA PET in the appropriate indications.

## Supplementary Information

**Additional file 1.**

**Additional file 2.**

**Additional file 3.**

## Data Availability

Not applicable.

## Abbreviations

ALARA: As low as reasonably achievable
BF: Biochemical Failure
CAPRA: Cancer of the Prostate Risk Assessment
CHUM: Centre Hospitalier de l’Université de Montréal
CI: Conventional Imaging
CT: Computed Tomography
CTCAE: Common Terminology Criteria for Adverse Events
DIL: Dominant Intraprostatic Lesion
DSMC: Data Safety Monitoring Committee
ECOG: Eastern Cooperative Oncology Group
EDC: Electronic Data Capture
EQ D2: Equivalent dose in 2Gy per fraction
FFS: Failure-Free Survival
GTV: Gross Target Volume
HDR: High Dose Rate
HT: Hormone Therapy
LDR: Low Dose Rate
LN: Lymph Node
MRI: Magnetic Resonance Imaging
PA: Para-aortic
PB: Prostate Bed
PCa: Prostate Cancer
PET: Positron Emission Tomography
PLND: Pelvic Lymph Node Dissection
PLNRT: Pelvic Lymph Node Radiotherapy
PSA: Prostate Specific Antigen
PSMA: Prostate Specific Membrane Antigen
QALY: Quality Adjusted Life Years
QoL: Quality of Life
RP: Radical Prostatectomy
RT: Radiotherapy
SABR: Stereotactic Ablative Radiotherapy
UCLA: University of California Los Angeles
UCSF: University of California San Francisco
