# Supplementary material for: PSMA PET/CT guided intensification of therapy in patients at risk of advanced prostate cancer (PATRON): a pragmatic phase III randomized controlled trial
Source: BMC Cancer. 2022 Mar 8;22:251. doi: 10.1186/s12885-022-09283-z (PMC8902723; doi:10.1186/s12885-022-09283-z)
Supplement: Supplementary file 1 — Additional file 1. [file 12885_2022_9283_MOESM1_ESM.pdf]

APPENDIX III - **Radiotherapy Standard of Care (SOC) Guidelines and QA Manual**

My signature below confirms that I have reviewed and approved this RT manual, and agree that it aligns with SOC practice at my institution. I will conduct this protocol as outlined therein, and according to Good Clinical Practice and all applicable local regulations.

Radiation Oncology Lead Investigator (Please Print)

Radiation Oncology Lead Investigator Signature

Date

1. Pelvic EBRT Guidelines - General

1.1 Equipment and Treatment Delivery

1.1.2 Equipment

Treatment is delivered using photons and an energy ranging from 6 MV to 18 MV (6MV preferable for lung SBRT). Flattening filter free beams are also allowed. Image-guidance based on CBCT imaging capabilities is required for linac-based delivery. Planning systems with capability for DICOM data transfer must be used. Cyberknife, which has a stereoscopic image guidance system and is a well-defined technology for SBRT, is permitted in this trial (see section 2). MRIdian and Unity (Elekta MR-LINAC) are also allowed.

1.1.2 Treatment Delivery

For MLC-linac-based delivery, static MLC, dynamic MLC, compensator IMRT, or VMAT are utilised. For Cyberknife technology, both cone-based and MLC-based deliveries are allowed.

1.1.3 Treatment Quality Assurance

Quality assurance will be conducted using a centralized imaging and dose-plan review system. All sites will be expected to identify a Radiation Oncology lead investigator who will serve as a contact and who will be provided with access. Prior to enrollment of patients on PATRON, sites will be required to download a standardized case of a salvage post-radical prostatectomy case with PSMA PET/CT identified pelvic lymph nodes. The test case will include CT Simulation and diagnostic PET/CT DICOM images. Centres will be required to plan a treatment with minimum dose targets of EQ2D 66Gy (a/b=3) to the prostate bed and PET positive nodes and EQD2 45Gy (a/b=3) to the PET negative regional nodes. The completed plan will be uploaded for review and once central review and approval (by the study PI or Radiation Oncology co-PI) then the site may commence accrual to PATRON (assuming all other study activation steps have been completed as well).

Real time case review will not be conducted. Sites will be responsible for uploading DICOM-RT files (Radiation Plan plus diagnostic PET/CT used for planning where applicable) for retrospective quality review at the time of analysis of the study.

1.2 **Positioning, Immobilization and Localization/Simulation**

1.2.1 Positioning and immobilization

In general, patients will be simulated and treated in the supine position with leg immobilization at a minimum, vac-loc or other pelvic immobilization devices are permitted. Patients must be in a position that they can maintain for the duration of each radiotherapy fraction.

1.2.3 Localization Imaging/Simulation

CT simulation of the pelvis is performed according to the institution's normal practice, including any specific instructions for bowel and bladder preparation for simulation and treatment. As a minimum, the scan volume should extend from at least L1/L2 to the lesser trochanters of the femurs.

IV or oral contrast can be administered, as clinically determined by the radiation oncologist or institutional policy for the body site to be treated. Organs affected may require a density-assignment equivalent to water for treatment planning.

Fusion of pelvic MRI for treatment planning is permitted; MRI should ideally be obtained the same day as planning CT simulation and in the treatment position. Recommended sequences include a T2-weighted axial MRI and diffusion weighted imaging with a b2000 acquisition. Some Centres may use a T1 pre and/or post-gadolinium axial MRI at their discretion. In these cases with MRI, the target can be contoured according to information on both the planning CT and MRI.

For those cases where PSMA PET/CT information is available, fusion of the PSMA PET/CT with the planning is required.

If artifacts are present, due to metal prostheses, the artifacts must be contoured and assigned a density of water. The metal implant must also be contoured and assigned an appropriate density.

1.3 **Motion Management**

All patients should be treated with a motion management strategy to account for inter-fraction target motion, at a minimum daily cone-beam CT (or equivalent), ideally with the aid of implanted fiducial markers. Intra-fraction motion management interventions (e.g. Calypso) are allowed depending on institutional capability.

1.4 **Volume Definitions**

1.4.1 Gross Tumor Volume (GTV)

The GTV is the disease that is visible on imaging if applicable. Each gross tumour should be contoured separately and named appropriately as per the nomenclature section below.

1.4.2 Clinical Target Volume (CTV)

The CTV is meant to encompass areas of microscopic disease that may be present around each GTV and/or areas of potential microscopic spread and is dependent on the body site that is being treated.

For the prostate, the primary CTV (CTVp) may consist of the prostate alone, the prostate and the proximal 1cm of seminal vesicles, or the prostate and the entire seminal vesicles. The CTVp may also include a margin of extraprostatic tissue abutting the GTV.

Where elective nodal irradiation is given, the appropriate nodal stations and balance of seminal vesicles will be contoured as per usual standard of care. This will comprise the nodal CTV (CTVn).

Elective nodal irradiation is at the discretion of the treating physician. Inclusion of regional pelvic lymph nodes is recommended in patients with:
(A) primary untreated high-risk prostate cancer or
(B) a high post-prostatectomy PSA (>0.3ngm/ml) at the time of salvage.

If performed it should follow the NRG Oncology Updated International Consensus Atlas on Pelvic Lymph Node Volumes for Intact and Post-Operative Prostate Cancer – 2020 (Hall et al. 2020). The Atlas is summarized as follows:

1. Commence contours at the bifurcation of the aorta into the **common iliac** arteries or the proximal inferior vena cava to the common iliac veins, whichever occurs more superiorly (typically at the level of L4-L5). Contour approximately 5-7 mm around each iliac vessel, including the entire circumference of both the iliac artery and vein. Bone, bowel, bladder, and muscle should be excluded from the nodal CTV contour. Where clinically indicated, CTV margins can be more generous, particularly anterior to vessels (10 mm). Ensure coverage posteriorly in the area formed between the psoas major and the vertebral body.

2. Include the **pre-vertebral, pre-sacral, and posterior mesorectal** nodes to the bottom of S3.

3. The posterior border of the CTV coming off the **internal iliac** vessels should extend to the anterior edge of the piriformis muscle, following the course of the pudendal artery and inferior gluteal artery.

4. The **external iliac** vessels' transition to the inguinal nodal region occurs when the external iliac vessels cross beneath the inguinal ligament into the inguinal canal. Examine for this transition, tapering off the external iliac nodes at that point. This should correspond to the entrance of the vascular structures into the inguinal canal, often best seen on the coronal images. The external iliac contours should typically end when the vessels are completely lateral to the most medial aspect of the acetabulum (near the mid-femoral head and fovea). At that point, the external iliac contribution of the CTVn contours should cease.

5. The CTVn attributed to the **obturator nodes** can be between 1-2 cm in width, extending to the posterior edge of the obturator internus muscle. Begin to taper the obturator nodes at the top of the seminal vesicles (or the top of the post-op

bed), extending approximately 1 cm anterior to the anterior edge of the obturator internus muscle. (MRI registration can be useful in this area) The obturator nodes should end where the seminal vesicles join the prostate, or approximately the midportion of the contoured post op CTV bed.

6. Exclude clearly peritoneal structures (ex. Bowel loops) as they are an anatomical barrier to lymphatic spread.

For postoperative cases, pathology and operative reports should be carefully considered in treatment volumes. Regions with pathologically involved nodes that exhibit extra-nodal tumor extension may have more generous CTVs. Surgical clips should be identified and potentially included at the discretion of the radiation oncologist. Altered lymph node spread is common, and larger volume expansions, including post-operative changes of uncertain significance may also be necessary.

Modifications when treating clinically node positive cases:

- Prophylactic nodal volumes (Prescribed EQD2 42-50Gy) should extend approximately 5-7 mm around clinically suspicious nodes. Involved pelvic lymph nodes do not have a CTV expansion. Residual (shrunken) gross nodes, post androgen deprivation therapy (ADT), should form the primary boost volume.

- If involved, the regional mesorectal LNs should be included. We recommend including mesorectal tissue in a 1cm isotropic expansion to the mesorectal LN GTV (i.e., GTVn_R_meso or GTVn_L_meso), making the volume continuous with the ipsilateral LN/obturator volume. The rectal lumen should be treated as an anatomical barrier.

- If involved, the paraortic lymph nodes should be included. We recommend extending the existing CTVn, as per the most recent NRG guidelines (Hall et al. 2020), continuously to and including the para-aortic region 1cm above the para-aortic LN GTV (i.e., GTVn_R_PA or GTVn_L_PA).

For the prostate bed, the primary CTV (CTVpb) includes the prostatic fossa and seminal vesicle remnants, if present, per the NRG Atlas (https://www.rtog.org/CoreLab/ContouringAtlases.aspx) and in accordance with the RTOG consensus guidelines (Michalski et al. 2010). https://www.ncbi.nlm.nih.gov/pmc/articles/PMC2847420/.

Briefly, the CTV should extend superiorly from the level of the caudal vas deferens remnant to >8-12 mm inferior to vesicourethral anastomosis (VUA). Below the superior border of the pubic symphysis, the anterior border extends to the posterior aspect of the pubis and posteriorly to the rectum, where it may be concave at the level of the VUA. At this level, the lateral border extends to the

levator ani. Above the pubic symphysis, the anterior border should encompass the posterior 1-2 cm of the bladder wall; posteriorly, it is bounded by the mesorectal fascia. At this level, the lateral border is the sacrorectogenitopubic fascia. Seminal vesicle remnants, if present, should be included in the CTV if there is pathologic evidence of their involvement.

1.4.4 Planning Target Volume (PTV)

The PTV for each body site to be treated is determined by each institution's policies and is dependent on many factors, including the type of immobilization used, the method of motion management used, and the type and accuracy of image guidance and set-up correction used. It is expected that each institution has measured the set-up errors and inter/intrafraction tumour motion for each body site and treatment technique. PTVs must be labelled as per the nomenclature section below. Daily image guidance is mandatory (ideally with a volumetric imaging technique such as CBCT).

For primary prostate radiotherapy, it is strongly recommended that at least 3 fiducial markers be inserted into the prostate to facilitate accurate daily image guidance. Typical PTV margins for treating the CTVp (prostate or prostate bed) would be 5-10mm.

If elective nodal irradiation is done, localization on treatment should be done to the fiducials in the prostate, followed by a review of the bone match. The PTV on the elective volume (including seminal vesicles and pelvic nodes) should be 5-10mm, to allow for some variability in position between bony anatomy and prostate fiducials.

1.4.5 Target Nomenclature

The GTV is the disease that is visible on imaging. Each gross tumour should be contoured separately and named appropriately. The GTV nomenclature detailed in Section 2.5.4 is highly recommended to identify different GTV's in various organ systems and nodal regions.
For the prostate gland, prostate bed, and optional elective pelvic nodal irradiation:
CTVp, PTVp: primary CTV comprised of prostate (+/- seminal vesicles)
CTVpb, PTVpb: primary CTV comprised of the prostate bed
CTVn, PTVpb: elective pelvic nodal CTV

Addition of other planning structures (e.g. PTV_66) as per local planning convention is allowed along as the planning structures above are included as a minimum.

1.4.6 Organs at Risk (OAR)

The critical normal structures in SOC prostate therapy are the bladder, rectum, femoral heads, and Bowel_bag. The normal tissues will be contoured and considered as solid organs.

Rectum: The rectum contour includes the rectum and anal canal and will be contoured on every slice from the rectosigmoid junction superiorly to the level of the ischial tuberosities inferiorly. Bowel preparation practices are at Centre discretion, at a minimum encouraging patient to evacuate their bowels (i.e. maintaining empty rectum) for treatment is recommended.

Bladder: The bladder contour will include the entirety of the bladder as defined on the simulation CT. The bladder should be moderately full (the patient should not be uncomfortable at simulation and probably will have more difficulty maintaining a full bladder during treatment).

Femur_LT, Femur_RT: The femoral heads should be contoured down to the region between the greater and lesser trochanters.

Bowel_bag: Small and large bowel in the pelvis and 1-2 cm superior to last slice of PTV.

1.5 **EBRT Dose Specification** (SOC only, please refer to protocol for intensification guidelines)

1.5.1 Targets

The prescription dose will be prescribed to the ICRU reference point for each target. Prescription can also be based on an isodose or to the median or mean dose to the PTV and recorded as such.

The aim is to have ≥ 95-99% of the PTV covered by 95% of the prescribed dose and ≥ 95-99% of the CTV/ITV covered by the prescription dose. Hotspots (105-110% of prescription dose) should be within the PTV and outside of OARs.

For presenting high-risk or salvage post RT, the following are considered SOC dose objectives (all represented in EQD2 with alpha/beta 1.4). Note that ultra-hypofractionated regimens are only considered SOC in select centers at this time, and require pre-approval at the time of credentialing.

PTVp – EQD2 (alpha/beta 1.4) = 78-92Gy
PTVn – EQD2 (alpha/beta 1.4) = 42-50Gy
PTVpb –EQD2 (alpha/beta 1.4) = 60-70Gy

SOC intensification of dose to gross visible disease on conventional imaging (PB recurrence, LNs, prostate tumor boost) is permitted within the dose ranges permitted in the study protocol (section 5.4.2).

1.5.2 Organs at Risk

Major – Considered a planning priority (compromise PTV coverage to respect)
Minor - Strive to achieve WITHOUT compromising PTV coverage

In general, for OARS situated well below deviation thresholds. , please apply the principle of As Low As Reasonably Achievable (ALARA) without compromising PTV coverage.

1.5.2.1 Sequential sum plans (final combined plan)

**(Gy)**	Rx 1.8-2Gy/Day		Rx 2.5Gy/Day		Rx 2.7Gy/Day		Rx 3Gy/Day	
	Major	Minor	Major	Minor	Major	Minor	Major	Minor
**Rectum**								
D50%	47	43	43	39	42	38	40	36
D25%	66	60	60	55	58	53	54	49
D15%	74	67	67	61	65	59	58	55
D0.5cc	78	76	71	69	68	66	60	59
**Bladder**								
D50%	50(65)	45(55)	46(60)	42(50)	45	41	42	38
D25%	66(68)	61(64)	60(65)	55(60)	58	54	55	50
D15%	76	70	69	63	66	61	60	57
**Bowel_bag**								
D200cc	48	44	44	41	43	40	40	37
D3cc	56	50	51	46	50	45	47	42
D0.1cc	66	58	60	53	58	51	54	48
**Femoral Heads** D1%	55	40	50	37	49	36	46	34

* The values in bracket are for RT salvage post-prostatectomy with prostate bed targets.

1.5.2.2 Single plans (concomitant)

(Gy)	5 fractions		15 fractions		20 fractions		22 fractions	
	Major	Minor	Major	Minor	Major	Minor	Major	Minor
**Rectum**								
D50%	18	16	37	33	40	36	41	37
D25%	25	23	50	45	54	49	56	51
D15%	28	26	55	50	58	55	63	57
D0.5cc	40	37	57	56	60	59	65	64
**Bladder** D2cc	39	38						
D50%	18	16	39	35	42	38	43	39
D25%	24	21	50	46	55	50	57	52
D15%	28	25	56	52	60	57	64	59
**Bowel_bag**								
D200cc	25	24	37	34	40	37	42	38
D3cc	29	26	43	38	47	42	48	43
D0.1cc	38	34	49	44	54	48	56	50
**Femoral Heads**								
D1%	28	22	42	32	46	34	47	35

Gy (post RP)	25 fractions		28 fractions		33 fractions		39 fractions	
	Major	Minor	Major	Minor	Major	Minor	Major	Minor
**Rectum**								
D50%	42	38	43	39	45	41	47	43
D25%	58	53	60	55	63	57	66	60
D15%	65	59	67	61	71	64	74	67
D0.5cc	68	66	71	69	74	72	78	76
**Bladder**								
D50%	45 (60)	41 (50)	46	42	48 (65)	44 (55)	50	45
D25%	58 (65)	54 (60)	60	55	63 (68)	58 (64)	66	61
D15%	66	61	69	63	72	66	76	70
**Bowel_bag**								
D200cc	43	40	44	41	46	42	48	44
D3cc	50	45	51	46	54	48	56	50
D0.1cc	58	51	60	53	63	56	66	58
**Femoral Heads**								
D1%	49	36	50	37	53	39	55	40

* The values in bracket are for RT salvage post-prostatectomy with prostate bed targets.

1.5.3 Fractionation

Fractionated RT is delivered on consecutive days Monday to Friday excluding weekend and statutory holidays.

1.5.4 Corrections for Interruptions

If delays occur in delivery, there are no corrections to be applied to the radiation dose delivered. The delay should be documented.

1.6 **Treatment Planning**

1.6.1 Beam Energy and Arrangement

6 MV to 18 MV is allowed. For IMRT, > 6 fields should be used and co-planar beam arrangement is preferable although adding non-coplanar beam angles is allowed if it will maximize the high dose conformity around the PTV. For VMAT, co-planar is preferable; non-coplanar arcs may be accepted. All Cyberknife beam arrangements are acceptable.

1.6.2 Isocentre Placement

When using a standard linac, the isocentre is defined as the common intersection point of gantry, collimator, and couch rotation for the treatment unit. For other types of treatment units (e.g., Tomotherapy or CyberKnife), a reference point in space that is typically positioned at the centre of the target is used instead of a mechanical isocentre.

1.6.3 Beam Modifiers

MLC are permitted for linacs. For Cyberknife,and Tomotherapy its own collimation (cones or MLC) system is used.

1.6.5 Planning Priorities and Compliance Criteria

The protocol priorities are: 1) OAR Major Criteria, 2) PTV coverage, 3) OAR Minor Criteria, 4) Unspecified tissues.

1.6.6 Inhomogeneity Corrections

Inhomogeneity corrections are recommended for final dose calculations.

1.6.8 Planning technique

Inverse planning technique should be used and calculation dose grid size must be equal to or less than 0.25 cm × 0.25 cm × 0.25 cm. All dose distributions shall include corrections for tissue heterogeneities. The approved algorithms to be used are found on the IROC Houston website (http://irochouston.mdanderson.org).

1.7 Verification

1.7.1 Position Verification/Correction

It is strongly recommended that prostate image-guidance be done using fiducials. At least three fiducials must be usable for tracking translation and rotation during treatment. If fewer than three fiducials can be tracked within the prostate, it is recommended that additional fiducials be placed, and patient replanned. Where possible, rotational corrections should be made.

1.8 Concurrent Therapy

Delivering radiotherapy with ADT (i.e., gonadotropin releasing hormone agonist or antagonist) is encouraged in a subset as detailed above. No concurrent chemotherapy, immunotherapy, additional theranostics, nor androgen-axis therapy (i.e., abiraterone acetate, enzalutamide, apalutamide, darolutamide) is to be delivered during radiotherapy nor planned in the adjuvant setting. Medications to manage acute side effects of radiotherapy can be delivered, including steroids, antiemetics, anti-diarrheals, and analgesics when appropriate.

1.9 Patient Specific Documentation Requirements

DICOM files (images, structures, plan and dose) should be submitted for review. This includes the planning CT and fusion image sets used for contouring. DICOM files will be uploaded to a centralized database (QIPCM) for review and archiving. Specific dose metrics will be recorded in the PATRON electronic database (CASTOR): anatomic region treated, dose to region, dose/fraction to region, treatment start and end dates, date of local QA and use of ancillary techniques (i.e. fiducials, SPACEOAR, 4DCT, respiratory motion)

1.10 Technical Considerations for HDR brachytherapy boost

HDR brachytherapy boost, in conjunction with EBRT, in high-risk patients is permitted at the discretion of the treating radiation oncologist. The implant may be performed during the EBRT portion of the treatment or within 2 weeks prior to its initiation or following its completion.

If a HDR brachytherapy boost is to be provided, patients will receive a single implant to provide a single HDR fraction of 15Gy, aiming for a minimum V100 of 90% to the prostate PTV. A boost to the GTV is permitted per boost guidelines detailed in protocol section 5.4.2.

The volume of bladder and rectum receiving 75% of the prescription dose must be kept to less than 1 cc (V75 <1 cc). The volume of urethra receiving 125% of the prescription dose must be kept to less than 1 cc (V125 <1 cc). The urethral V150 should be 0 cc (V150 = 0 cc). The volume of rectum receiving 75% of the prescription should be kept to less than 1cc.

1.11 Technical Considerations for LDR brachytherapy boost.

LDR brachytherapy boost, in conjunction with EBRT, in high-risk patients is permitted at the discretion of the treating radiation oncologist. The implant may be performed during the EBRT portion of the treatment or within 2 weeks prior to its initiation or following its completion. Pre-planning or intra-operative planning is permitted as is stranded or loose I125 seeds. LDR boost will be as per ASCENDE-RT protocol.

Patients receiving an LDR will be treated with a 125-Iodine brachytherapy boost prescribed to a minimal peripheral dose (MPD) of 115 Gy. For the LDR boost, GTV = CTV and include the entire TRUS-visualized prostate. The PTV included a 3 to 5 mm margins on the CTV in all directions except posterior where no margin is added. Modified peripheral with urethral sparing was specified and LDR-PB was performed as a day-care procedure under general or spinal anesthesia.

A post implant CT scan with four weeks after the procedure is required, and post-implant dose metrics (D90, V100, and V150) calculated. Additional radiotherapy (2nd implant or additional external beam radiotherapy) is recommended if the D90 is <90% of the prescription MDP or the V100 <75%. Independent review of implant quality is not required.

2. EBRT Guidelines - SBRT for oligometastases

2.1 Equipment and Treatment Delivery

2.1.2 Equipment

Treatment is delivered using photons and an energy ranging from 6 MV to 18 MV (6MV preferable for lung SBRT). Flattening filter free beams are also allowed. Image-guidance based on CBCT imaging capabilities is required for linac-based delivery. Planning systems with capability for DICOM data transfer must be used. Cyberknife, which has a stereoscopic image guidance system and is a well-defined technology for SBRT, is permitted in this trial. MRIdian and Unity (Elekta MR-LINAC) are also allowed.

2.1.2 Treatment Delivery

For MLC-linac-based delivery, static MLC, dynamic MLC, compensator IMRT, or VMAT are utilised. Motion management during SBRT is required for mobile tumours like lung and liver as per institutional practice. For Cyberknife technology, both cone-based and MLC-based deliveries are allowed. For thoracic and upper abdominal tumour targets, motion management is mandatory. This could be done with 4D-CT simulation with or without abdominal compression and generation of an ITV. Alternatively, respiratory gating or breath hold techniques are permitted.

2.1.3 Treatment Quality Assurance
Quality assurance for SBRT will be by virtue of credentialing for other SBRT trials as specified in section 9.2. All sites will also have been required to complete the test case as described in section 1.1.3. Real time review of SBRT plans will not be conducted. Sites will be required to upload DICOM RT or DICOM imaging files for patients treated for oligometastatic disease, in order to facilitate subsequent retrospective review (Section 1.1.3).

2.2 **Positioning, Immobilization and Localization/Simulation**

1.2.1 Positioning

In general, patients will be simulated and treated in the supine position, ideally with arms positioned away from the treatment area (e.g. above their head for thoracic/upper abdominal targets). Prone positioning is allowed for SBRT of oligometastases, if the prone position facilitates favorable position of the Organs at Risk relative to the tumour volume and in comparison to the supine position. Patients must be in a position that they can maintain for the duration of each radiotherapy fraction.

2.2.2 Immobilization

For spine SBRT, patients will be in a body immobilization device for tumours on and below the 5th thoracic (T5) vertebrae, and in a thermoplastic head and neck immobilization mask system for tumours extending from the 1st cervical vertebrae (C1) to the 4th thoracic vertebrae (T4).

For all other sites, the body immobilization device will be according to institutional departmental policy.

In the case of Cyberknife treatments, body immobilization is not mandatory due to the near realtime image guidance verification system inherent to Cyberknife. However, for MLC-linac or Tomotherapy based therapy, immobilization is mandatory.

2.2.3 Localization Imaging/Simulation

CT simulation of the body site(s) to be treated is performed according to the institution's normal practice for EBRT and SBRT. The scan volume should include the tumour target and organs at risk are deemed necessary to develop a high quality radiotherapy plan. As a minimum, the scan volume should extend at least 5 cm above and below the PTV, and inclusion of entire relevant organs at risk should be performed (i.e. entire lungs or entire liver).

Consultation with physics and therapy in advance of CT Simulation to ensure an appropriate planning strategy is strongly advised for patients with complex oligometastatic anatomies (i.e. proximity to previously irradiated structures, distribution between mobile and non-mobile locations, close proximity between lesions)

A 3-D treatment planning volumetric imaging study is to be used to define the target volumes and organs at risk. Planning CT slice thickness of ≤ 3 mm is required (axial image size 512 x 512). Ideally, one CT scan should be used to contour all areas of disease for this protocol so that composite dose distributions/plans can be generated to document total doses from multiple treatment sites. However, it is recognized that this may not be possible if treatment of different body sites requires different scanning techniques (e.g. 4DCT or static CT), patient positioning (e.g. arms up or down) or immobilization devices, or if there is large spatial separation between tumours. For these cases, multiple CT scans are allowed. However, the number of scanned tumours to be treated should be maximized in each CT scan so that a composite dose distribution including as many tumours as possible can be created.

IV or oral contrast can be administered, as clinically determined by the radiation oncologist or institutional policy for the body site to be treated. Organs affected may require a density-assignment equivalent to water for treatment planning.

For selected sites (such as prostate gland, prostate bed with gross recurrence, bone, spine, and liver), MRI for fusion to the planning CT should be obtained, preferably with the patient in the simulation position. The sequences include a T2-weighted axial MRI and some centres may use a T1 pre and/or post-gadolinium axial MRI at their discretion. In these cases with MRI, the target can be contoured according to information on both the planning CT and MRI. For spine, MR fusion is mandatory for target and spinal cord/thecal sac contouring.

If artifacts are present, due to metal prostheses, the artifacts must be contoured and assigned a density of water. The metal implant must also be contoured and assigned an appropriate density.

2.3 **Motion Management**

All metastases with potential for respiratory motion (thoracic and upper abdominal tumours) should be evaluated by appropriate means including 4D CT scan, implanted fiducial marker, or fluoroscopy at the time of simulation. Institutions are required to

have a commissioned respiratory motion management (RMM) solution to accommodate treatment of lesions with large respiratory motion (i.e. >1.0cm). Such solutions should be able to integrate/accommodate the treatment of lesions that do and do not require RMM within the same patient.

2.4 **Volume Definitions**

In this protocol, participants on the intervention arm may receive treatment to up to 5 metastases in addition to a single plan which treat the prostate and pelvic lymphatics. Thus, there may be up to 6 separate radiotherapy plans for participants on the intervention arm. For all radiation plans, GTV and PTV contours are mandatory per target treated. CTV is optional, depending on the tumour site being treated. Where applicable, the ITV will be specified. For nonspine targets (e.g. liver, lung), the PTV margin is based on institutional practice.

2.4.1 Gross Tumour Volume (GTV)

The GTV is the disease that is visible on imaging if applicable. Each gross tumour should be contoured separately and named appropriately according to a uniform nomenclature such as naming based on the organ involved, and numbered from cranially to caudally. For example, in a patient with 3 lung lesions, there would be: GTV_lung_1, GTV_lung_2, and GTV_lung_3, and corresponding PTV_lung_1, PTV_lung_2_, and PTV_lung_3, representing the lesions from superior to inferior.

If 4D-CT is used, contours done on various phases of respiration should be labeled accordingly after a caret ("^") by providing details of the respiratory phase used to generate the images, including FB = free breathing, Avg = average, 0 = end inhalation, 50= end expiration, MIP = maximum intensity projection (eg GTV_Kidney^0, GTV_Kidney^MIP)

If the targets are contoured on more than 1 image set, a label to designate the treatment modality should be added after an underscore, including CT = computed tomography, PT = positron emission tomography, MR – magnetic resonance imaging (e.g. GTV_Spine_MR, GTV_Spine_CT). Where more than 1 image set is used for any one treatment modality (e.g. T1 and T2 MR images), an Arabic number will be added (e.g. GTV_Spine_MR1 and GTV_Spine_MR2).

Labelling of other CTVs, ITVs, and PTVs should follow the same guidelines listed above within 2.4.1.

2.4.2 Clinical Target Volume (CTV)

The CTV is meant to encompass areas of microscopic disease that may be present around each GTV and/or areas of potential microscopic spread and is dependent on the body site that is being treated.

It is recommended that there will be no CTV expansion around the GTV, (i.e. GTV = CTV) for most metastatic tumor sites with the exception of spine SBRT. However, a CTV expansion of no more than 1cm is allowed for tumours if the physician is not confident about the boundaries of the GTV. For spine SBRT, the CTV will be based on anatomic boundaries as described below. CTV's must be labelled as per the nomenclature section below.

2.4.2.1 Spine Specific CTV delineation

The International Spinal Consortium Guideline is a reference for CTV delineation and adherence to that approach is recommended. (Dunne et al 2019)

For the spine, an anatomic approach is taken to the CTV based on where the lesion is located within the spinal segment. The rules for CTV are as follow:

1. If the vertebral body is involved with GTV, then the entire vertebral body is taken as CTV.

2. If the ipsilateral pedicle and/or lamina, and/or transverse process have GTV, then the entire ipsilateral posterior segment (pedicle, lamina and transverse process) is taken into the CTV. The inclusion of the spinous process as CTV is at the radiation oncologist's discretion.

3. If bilateral involvement of the pedicle, and/or lamina and/or transverse process with GTV, then the entire posterior segment anatomy is taken into the CTV. Inclusion of the spinous process as CTV is at the discretion of the radiation oncologist and recommended if bilateral lamina are involved.

4. If the spinous process is involved with GTV alone, then the bilateral lamina are to be taken into the CTV.

In the case of epidural disease, a 5 mm anatomic margin (excluding the spinal cord) beyond the GTV is required within the epidural compartment including in the cranio-caudal direction. A circumferential CTV as per a donut based CTV is allowed and encouraged in the case of epidural disease at the discretion of the treating radiation oncologist. If paraspinal disease is present, a minimum of 5 mm CTV margin must be applied beyond the GTV. The spinal cord is always excluded from GTV and CTV.

2.4.3 Internal Margin (IM)

If 4D-CT simulation is used for thoracic and upper abdominal tumours that move with breathing, an ITV will be generated by combining the GTV/CTV volumes from the various 4D-CT bins. As a minimum, the GTV/CTV should be contoured on the end inspiratory and end expiratory bins of the 4DCT, although additional bins can be used to generate the appropriate ITV. For peripheral lung tumours surrounded by low density

lung, the maximum intensity projection (MIP) along with the end inspiratory and end expiratory bins from the 4D-CT may be used to generate the ITV. ITVs must be labelled as per the nomenclature section below.

2.4.4 Planning Target Volume (PTV)

The PTV for each body site to be treated is determined by each institution's policies and is dependent on many factors, including the type of immobilization used, the method of motion management used, and the type and accuracy of image guidance and set-up correction used. It is expected that each institution has measured the set-up errors and inter/intrafraction tumour motion for each body site and treatment technique. PTVs must be labelled as per the nomenclature section below.

Daily image guidance is mandatory (ideally with a volumetric imaging technique such as CBCT).

PTV margins for non-spine metastases should be 5 mm, but a minimum of 3 mm can be used if the institution has the experience with smaller margins.

For the spine, a uniform margin of 1 to 3 mm is required for PTV. The specific margin will be according to centre/department policy but no more than 3 mm and no less than 1 mm are permitted. In the case where the target vertebrae includes adjacent levels, each target vertebrae requires a GTV and CTV. The PTV margin must also be applied to the target vertebrae CTV in order to report dosimetric data for each target; however, the PTV to be planned upon is the multilevel PTV. In the case of non-contiguous levels, then the approach is to plan on individual PTVs.

2.4.6 Organs at Risk (OAR)

The OARs for each body site will be contoured according to each institution's treatment policies, although the nomenclature and dose specification sections below will provide guidelines. PRVs should be contoured according to each institution's routine practice, although it is not required for this protocol with the exception of spine SBRT.

2.4.6.1 Spine specific SBRT OAR

1. Spinal cord (SpinalCord): contoured based on the diagnostic CT or T1 and/or T2 MRI fused to the planning CT. A 1.5 to 2mm margin for PRV is to be applied. The thecal sac (PRV015_SpinalCord) can also be contoured.

2. Cauda equina (CaudaEquina): The thecal sac (ThecalSac) must be contoured as a surrogate for the cauda equina when relevant to the spinal segment to be treated.

3. Kidneys (Kidney_L, Kidney_R)): each kidney will be contoured when relevant to the spinal segment to be treated based on the CT scan.

4. The Larynx, Pharynx, Esophagus, Trachea, Stomach, Liver, Rectum, Bowel_Small, and Bowel_Large should be contoured based on the CT where relevant to the spinal segment to be treated.

5. For sacral tumours (S1-S5), Nerve_Roots should be contoured and followed until the point they reach abdominal contents.

6. PRV margin of 1.5-2 mm is required only for the spinal cord for spine SBRT, with a dose limit of 17 Gy as a point maximum applied to this structure for the 2 fraction SBRT. In 5 fractions, a 25 Gy point maximum dose limit is allowed. If the thecal sac is also contoured, then the dose limit to the spinal cord PRV is the dose limiting OAR. For cauda equina, the thecal sac is contoured and no PRV is applied, with the dose limit applied to the thecal sac itself.

2.4.6.2 Non-spine SBRT OAR

Labelling of OARs for all other sites should follow the guidelines suggested by AAPM Report No. 263. (see table below)

ACJoint	Ear_Internal_L	Kidney_L	Pancreas^0
A_Aorta	Ear_Internal_R	Kidney_L^0	Pancreas^50
A_Carotid_L	Esophagus	Kidney_L^50	Parotid_L
A_Carotid_R	Eye_L	Kidney_R	Parotid_R
AntChamber	Eye_R	Kidney_R^0	PenileBulb
Anus	Femur_L	Kidney_R^50	Pharynx
Bladder	Femur_R	Larynx	Prostate
Bladder_Wall	FemoralHead_L	Lens_L	PRV015_SpinalCord/Canal
Bone_Mandible	FemoralHead_R	Lens_R	PRV05_SpinalCord/Canal
Bone_Pelvic	Gallbladder	Lips	PRV03_Brainstem
Bowel	Genitals	Liver	PRV05_Brainstem
Bowel_Large	Glnd_Lacrimal_L	Liver^0	Rectum
Bowel_Small	Glnd_Lacrimal_R	Liver^50	Rectum_Wall
BrachialPlexs	Glnd_Submand_L	Liver^Uninv	Ribs
Brain	Glnd_Submand_R	Liver-GTV	SeminalVes
Brainstem	Glnd_Thyroid	Liver-ITV	Skin
Bronchus_Large	GreatVes	Lung_L	Spc_Bowel
Bronchus_Small	Heart	Lung_R	SpinalCanal
CardiacVes	Hippocampus_L	Lungs	SpinalCord
CaudaEquina	Hippocampus_R	Lungs-GTV	Stomach
Cavity_Oral	IMCVes	Lungs-CTV	ThecalSac
Chestwall	Kidney_Cortex_L	Lungs-ITV	Trachea
Cochlea_L	Kidney_Cortex_R	Nerve_Roots	TracheaMainBronchus
Cochlea_R	Kidney_Cortex^Uninv	OpticChiasm	Urethra
Colon	Kidney_Cysts	OpticNrv_L	Uterus
Cricoid	Kidney_Hilum_L	OpticNrv_R	V_Venacava_I
Duodenum	Kidney_Hilum_R	Pancreas	VocalCords

2.5 **SBRT Dose Specification** (SOC only, please refer to protocol for intensification guidelines)

2.5.1 Targets

The prescription dose will be prescribed to the ICRU reference point for each target. Prescription can also be based on an isodose or to the median or mean dose to the PTV and recorded as such. Effort should be made to develop highly conformal plans with the high dose volume shaped around the PTV(s).

2.5.2 Organs at Risk

OAR dose limits will adhere to institutional practice and guidelines. For multiple targets in the same body site, composite plans should be calculated if feasible. The tables below represent guidelines for suggested OAR dose limits. Doses to OARs should be minimized, especially in the treatment of multiple targets. Furthermore, OAR Dose limits must be respected in all cases, with compromise of PTV target coverage if needed.

OAR Dose Limits for 2 fraction SBRT guidelines (per PR20):

**Serial Tissue**	**Max Point Dose (Gy)**	**Mean Dose (Gy)**
Spinal cord plus PRV/thecal sac for spine SBRT	17	--
Esophagus	20	--
Trachea	20	--
Pharynx	20	8-10
Larynx	20	8-10
Stomach	20	--
Small bowel	20	--
Large bowel	20	--
Rectum	20	--
Nerve roots	26	--
**Parallel Tissue**	**Critical Volume (cc)**	**Critical Volume Dose Max (Gy)**
Parotids	Mean Dose	5-10
Lung (Right & Left)	< 3%<br>< 10%<br>< 35%<br>Mean dose	20-22<br>10-12<br>5-7<br>5-8
Liver	Max dose<br>Mean dose	24<br>8-10
Renal cortex (Right & Left)	Max dose<br>Mean dose	24<br>5-7

OAR Dose Limits for 5 fraction SBRT guidelines (for non-pelvic targets, per PR20):

Serial Tissue	Volume	Volume Max (Gy)	Max Point Dose (Gy)
Spinal Cord/CaudaEquina contoured per spinal canal	< 0.35 cc<br>< 1.2 cc	23<br>14.5	30
Spinal cord plus PRV/thecal sac for spine SBRT	Max Point dose	25	25
Esophagus	< 5 cc	27.5	38
Brachial Plexus	< 3 cc	30	32
Heart/Pericardium	< 15 cc	32	53
Great vessels	< 10 cc	47	53
Trachea and Large Bronchus	< 4 cc	18	53
Bronchus- smaller airways	< 0.5 cc	21	33
Rib	< 1 cc	35	43
Skin	< 10 cc	36.5	39.5
Stomach	< 5 cc	25	38
Small bowel	< 5 cc<br>< 10 cc	25<br>12.5	38
Large bowel	< 20 cc	25	40
Rectum	< 20 cc<br>20%<br>1cc<br>2cc	25<br>29<br>36<br>35	
Bladder	< 40%<br>< 10%<br>< 10cc	18.1<br>32<br>37	
Penile Bulb	< 3 cc	30	50
Femoral Heads (Right & Left)	< 10 cc	30	
Renal hilum/vascular trunk	< 2/3 volume	23	
**Parallel Tissue**	**Critical Volume (cc)**	**Critical Volume Dose Max (Gy)**	
Lung (Right & Left)	1500 cc<br>1000 cc	12.5<br>13.5	
Liver	700cc	21	
(Liver-ITV)-15Gy isodose volume	700cc	15	
Renal cortex (Right & Left)	200 cc<br>35%	17.5<br>18	

2.5.3 Fractionation

SBRT may be delivered on consecutive days Monday to Friday excluding weekend and statutory holidays, or alternating days, or an alternative schedule approach that is defined within the institution.

2.5.4 Corrections for Interruptions
If delays occur in delivery, there are no corrections to be applied to the radiation dose delivered, but the delay should be documented.

2.6 **Treatment Planning**

2.6.1 Beam Energy and Arrangement

6 MV to 18 MV is allowed. For IMRT, > 6 fields should be used and co-planar beam arrangement is preferable although adding non-coplanar beam angles is allowed if it will maximize the high dose conformity around the PTV. For VMAT, co-planar is preferable but non-coplanar arcs may be accepted. All Cyberknife beam arrangements are acceptable.

2.6.2 Isocentre Placement

When using a standard linac, the isocentre is defined as the common intersection point of gantry, collimator, and couch rotation for the treatment unit. For other types of treatment units (e.g., Tomotherapy or CyberKnife), a reference point in space that is typically positioned at the centre of the target is used instead of a mechanical isocentre.

When treating multiple lesions multiple isocentres can be used, each centered on a separate lesion to treat different targets on different days in order to decrease treatment time for a single day. For widely spaced lesions (over 10 cm apart), localization is improved when the isocentre is placed in the centre of each target and image-guidance is performed individually for each target. This is due to the limitation of most IGRT systems, which ignore necessary rotational corrections when table shift coordinates are derived. Some platforms, including Cyberknife and Tomotherapy, are inherently non-isocentric. These platforms take special account in the setup and treatment process to rigorously detect and account for rotations to avoid errors, making them exempt from the separate isocentres for each lesion requirement.

2.6.3 Beam Modifiers

MLC are permitted for linacs. For Cyberknife, its own collimation (cones or MLC) system is used.

2.6.5 Planning Priorities and Compliance Criteria

The protocol priorities are: 1) OAR, 2) PTV coverage, 3) Unspecified tissues.

PTV coverage should aim for a minimum PTV V95>95%.

2.6.6 Inhomogeneity Corrections

Inhomogeneity corrections are recommended for final dose calculations.

2.6.7 Acceptable Dose Heterogeneity

For non-spine SBRT, the aim is to have ≥ 95-99% of the PTV covered by 95% of the prescribed dose and ≥ 95-99% of the CTV/ITV covered by the prescription dose.

In general, dose homogeneity (minimizing hotspots within the PTV) is desired for the SBRT plans, with the exception of spine SBRT.

Hotspots (> 105-110% of prescription dose) should be within the PTV and preferably away from adjacent OARs.

High dose conformity: Effort should be made to keep the conformity index (volume of prescribed % isodose to the PTV/volume of PTV) ≤ 1.2-1.3 for SBRT plans. Higher values are acceptable if the high dose spillage outside the PTV is deemed to be acceptable by the radiation oncologist.

For spine SBRT, the aim is to achieve coverage of the PTV by at least 80% of the prescribed dose and to achieve this, 80% of the CTV should get 95-100% of the prescribed dose. A dose heterogeneity of +50% is allowed in the PTV.

2.6.8 Planning technique

Inverse planning technique should be used and calculation dose grid size must be equal to or less than 0.25 cm × 0.25 cm × 0.25 cm. All dose distributions shall include corrections for tissue heterogeneities. The approved algorithms to be used are found on the IROC Houston website (http://irochouston.mdanderson.org).

Composite plans should be generated internally when possible to incorporate the dose to surrounding normal tissues from each metastasis treated. The composite doses to critical normal tissues will be used to evaluate plan compliance according to departmental protocol. Composite planning refers to dose summation from multiple treatment sites on a single CT scan that encompasses the relevant anatomy. Composite treatment planning is best accomplished by obtaining a planning CT dataset that incorporates all targets and relevant critical structures in the imaging study. If this is not possible due to restrictions on the size of the imaging study that can be managed by the treatment planning system, CT datasets should be divided into two parts and treatment fields should be adjusted so that dose spillage from the treatment of targets in one dataset to the next is minimal such that the dose contributions do not require summation. The datasets should be obtained so that they have some amount of overlap that can be used to fuse the information using a rigid registration technique.

The use of deformable registration to sum dose is recommended if the centre is using a system regularly with an appropriate deformable registration QA in place. In general, it is best to perform CT scanning with the patient in the same position. This implies that, for example, all lesions planned on a gated CT scan must be treated with gating.

1.6.9 Treatment Delivery Constraints

No minimum segment size will be specified, but each centre should follow departmental policy according to the already existing clinical SBRT protocol. The treatment planning system beam model must have been commissioned such that the monitor units delivered by the minimum segment size can be calculated with acceptable accuracy against measurements. The number of segments per field should be minimized for IMRT (total of 60-90 segments for IMRT plan is reasonable). For VMAT, arc span should be adjusted according to position / laterality of target.

2.7 Verification

2.7.1 Position Verification/Correction

Ideally, soft tissue tumour or fiducial marker matching should be performed using CBCT for linac based treatment. As a minimum, bony anatomy or other surrogates of tumour position (like diaphragm) can be used for matching if the tumour is not visible. If the treatment time is excessively long (according to centre protocol), or there is suspicion that the patient position may have changed, intra-fractional verification imaging to check patient/tumour position can be done.

It is strongly recommended that prostate image-guidance be done using fiducials. At least three fiducials must be usable for tracking translation and rotation during treatment. If fewer than three fiducials can be tracked within the prostate, it is recommended that additional fiducials be placed, and patient replanned. Where possible, rotational corrections should be made.

The Cyberknife uses its own stereoscopic imaging system and verification imaging should be performed prior to all fractions.

2.8 Concurrent Therapy

No concurrent chemotherapy or immunotherapy is to be delivered during radiotherapy. Delivering radiotherapy with ADT, including second generation hormone therapy agents, is allowed and encouraged. Medications to manage acute side effects of radiotherapy can be delivered, including steroids, antiemetics, anti-diarrheals, and analgesics when appropriate.

For patients receiving radiotherapy to the abdominal areas, prophylactic anti-emetics is allowed.

2.9 Patient Specific Documentation Requirements

DICOM files (images, structures, plan and dose) should be submitted for review. This includes the planning CT and fusion image sets used for contouring. For 4DCT, it is

sufficient to send only the end-inspiration and end-expiration phases. DICOM files will be uploaded to a centralized database (QIPCM) for review and archiving. Specific dose metrics will be recorded in the PATRON electronic database (CASTOR): anatomic region treated, dose to region, dose/fraction to region, treatment start and end dates, date of local QA and use of ancillary techniques (i.e. fiducials, SPACEOAR, 4DCT, respiratory motion)
