# Supplementary material for: PSMA PET/CT guided intensification of therapy in patients at risk of advanced prostate cancer (PATRON): a pragmatic phase III randomized controlled trial
Source: BMC Cancer. 2022 Mar 8;22:251. doi: 10.1186/s12885-022-09283-z (PMC8902723; doi:10.1186/s12885-022-09283-z)
Supplement: Supplementary file 2 — Additional file 2. [file 12885_2022_9283_MOESM2_ESM.pdf]

APPENDIX II

Charlson Comorbidity Index

1. Scoring: Comorbidity Component
    1. Myocardial Infarction (1 point)c
    2. Congestive Heart Failure (1 point)
    3. Peripheral Vascular Disease (1 point)
    4. Cerebrovascular Disease (1 point)
    5. Dementia (1 point)
    6. COPD (chronic obstructive pulmonary disease) (1 point)
    7. Connective Tissue Disease (1 point)
    8. Peptic Ulcer Disease (1 point)
    9. Diabetes Mellitus (1 point uncomplicated, 2 points if end-organ damage)
    10. Moderate to Severe Chronic Kidney Disease (2 points)
    11. Hemiplegia (2 points)
    12. Leukemia (2 points)
    13. Malignant Lymphoma (2 points)
    14. Solid Tumor, excluding prostate cancer (2 points, 6 points if metastatic)
    15. Liver Disease (1 point mild, 3 points if moderate to severe)
    16. AIDS (not just HIV positive) (6 points)

2. Scoring: Age
    1. Age <50 years: 0 points
    2. Age 50-59 years: 1 point
    3. Age 60-69 years: 2 points
    4. Age 70-79 years: 3 points
    5. Age >80 years: 4 points
