# Supplementary material for: PSMA PET/CT guided intensification of therapy in patients at risk of advanced prostate cancer (PATRON): a pragmatic phase III randomized controlled trial
Source: BMC Cancer. 2022 Mar 8;22:251. doi: 10.1186/s12885-022-09283-z (PMC8902723; doi:10.1186/s12885-022-09283-z)
Supplement: Supplementary file 3 — Additional file 3. [file 12885_2022_9283_MOESM3_ESM.pdf]

FUM-8038

APPROUVÉ – CÉR CHUM

DATE 14 mai 2021
INITIALES: MJB

INFORMATION AND CONSENT FORM

**Project title:**	**PSMA PET/CT GUIDED INTENSIFICATION OF THERAPY IN PATIENTS AT RISK OF ADVANCED PROSTATE CANCER (PATRON)**
**Principal researcher:**	Dr. Cynthia Ménard, MD, Radiation oncologist, CHUM
**Co-researchers**:	Dr. Fred Saad, MD, Urologist-oncologist, CRCHUM<br>Dr. Guila Delouya, MD, Radiation oncologist, CHUM<br>Dr. Jean DaSilva, PhD, Radiochemistry, CRCHUM<br>Dr. Daniel Juneau, MD, Nuclear Medecine, CHUM
**Funding:**	Canadian Cancer Society
**Collaborator:**	Lantheus Medical Imaging (providing radiotracer)
**Multicenter identifier:**	MP-02-2021-9258
**Project number at CHUM:**	20.208

PREAMBLE

You are being invited to take in a research project because you have prostate cancer that will be treated with radiation or surgery, and you are at risk of advanced disease. Before agreeing to take part in this project and to sign this informed consent form, please take your time to read and consider carefully the following information.

This form may contain terms which you do not understand. This form may contain terms which you do not understand. Please ask the principal investigator or other members of the research team any questions you feel might be helpful and to explain any word or information which is not clear to you.

NATURE AND OBJECTIVES OF THE PROJECT

In order to measure the spread of prostate cancer, oncologists routinely use an imaging test called a CT-scan, as well as a bone scan. Unfortunately these tests are not perfect.

To improve the detection of prostate cancer, researchers will study a radiotracer (slightly radioactive contrast agent), which binds to a protein called prostate-specific membrane antigen (PSMA). More PSMA is found in prostate cancer tissue than in normal tissue. This radiotracer will be used in this study to perform a PET scan. Compared to a standard CT-Scan, PSMA-PET can better detect the spread of prostate cancer. The results of the PSMA-PET scan are provided to the treating doctor who can then adjust treatment as needed. If new lesions are detected with a PSMA-PET scan, they are in generally treated at the moment of surgery or radiotherapy.

Prior studies have shown that PSMA-PET scans can detect spread of prostate cancer to lymph nodes, bones, or other parts of the body that other types of scans cannot detect. Better knowledge of the site of cancer spread may help in the fight against prostate cancer by directing surgery or radiotherapy, but this potential has not yet been shown.

The PSMA radiotracer under study is already used and deemed safe in many countries in Europe to guided treatment of prostate cancer. In Canada, this product is considered experimental and can only be used in the context of research for now. Health Canada has however provided authorisation for use in this study.

In this study, researchers will compare a sub-group of men receiving standard surgery or radiotherapy guided by standard imaging tests (Group 2) to a sub-group of men where the treatment is also guided by PSMA-PET (Group 1). Researchers think that treatment guided by PSMA-PET will help in the fight against prostate cancer and could improve quality of life in the long term. However, given that PSMA-PET detects more sites of cancer, the treatment may be more intense and could lead to more side effects.

The principal objective of this study is therefore to determine if intensified therapy guided by PSMA-PET improves the effectiveness of cancer treatment in a cost effective way, including any positive or detrimental impact of treatment on patients' wellbeing, and if the advantages outweigh the risks.

NUMBER OF STUDY PARTICIPANTS AND LENGTH OF THE PARTICIPATION

Seven hundred and seventy six (776) men from many hospitals across Canada will participate in this study, with 250 at CHUM.

The total duration of the study is 8 years. Your participation in this study will correspond to the duration of your treatment and follow-ups for a total duration of 5 to 8 years.

NATURE OF PARTICIPATION REQUESTED

If you accept to participate in this study, and after signing the present consent form, you will be randomly assigned (by chance like flipping a coin) by a computer program to one of two groups as follows:

- **Group 1 (Experimental Treatment):** ***Intensified treatment guided by PSMA-PET***

If you are assigned to Group 1, you will undergo a PSMA-PET scan with the radiotracer under study. After this scan, if new lesions are found, your treatment will be adjusted to better treat the detected lesions. If no new lesion is detected, your treatment will proceed as planned in standard care.

- **Group 2 (Standard Treatment):** ***Treatment without PSMA-PET***

If you are assigned to Group 2 your treatment will proceed as planned.

You are equally likely to be assigned to one or the other group of the study. Neither you nor your doctor can choose to which group you will be assigned.

If you agree to participate in this study, you also accept to receive other tests and procedures described in the section ¨Study related procedures¨ below.

STUDY RELATED PROCEDURES

You will find a table of visits and study procedures at the end of this consent form.

The study will include three (3) phases:

1) Prior to treatment phase
2) Treatment phase
3) Follow-up phase

1) Prior to treatment phase

- Group1 PSMA-PET (1 visit, 2-4 hours)

- Intravenous injection of the radiotracer: The tracer will be absorbed in certain regions of your body and will be detected by the PET scanner. You will be asked to drink at least 2 glasses of water during the waiting period between the injection of radiotracer and the imaging test.
- Image acquisition: roughly 90 minutes after the injection of PSMA radiotracer, you will undergo an imaging test with a PET scanner to detect prostate cancer cells. During the scan, you will lie on your back still for roughly 20 minutes or more if the images need to be repeated (for example if the radiotracer in your urine is degrading the quality of the images). In this case, we may ask your to drink 1-2 more glasses of water and we will inject a diuretic (a medicine that makes your urinate). The diuretic used will be Lasix (Furosemide). We will make sure that this medicine is safe for you based on your medical history.
- **A special preparation is needed prior to the PET scan, and precaution are needed after the scan**

Before the PET scan:

- You must **<u>drink at least 2 glasses of water</u>** after the injection and before image acquisition.
- You must **void as often as needed and empty your bladder** right before the imaging test.

After the PET scan:

- You are recommended to drink water (about 250 mL) to clear the radiotracer from your body and void often.

- The PET images acquired will be reviewed by a nuclear medicine specialist and the results will be shared with your treating physician.
- In some cases a different imaging test called a magnetic resonance image (MRI) or a biopsy, which is not part of standard tests, may be needed to clarify the results of the PET scan.

<u>- Groups 1 and 2 (all participants) (1 visit, 1 hour)</u>

- A blood test (5mL, or 1 teaspoon) to measure your PSA if a recent measurement is not available.
- A targeted medical examination based on your medical history and current symptoms.
- A review of your current medications.
- Three (3) surveys to complete by internet regarding your general wellbeing, as well as your urinary, intestinal, sexual, and hormonal symptoms before treatment. Each survey will take approximately 5-10 minutes to complete.

2) Treatment phase (1-38 visits)

<u>- Group 1 PSMA-Pet (0 to 6 visits, 1 hour each)</u>

Your doctor will adjust your treatment according to the results of the PSMA-PET based on the following scenarios:

- If no new lesions are found, your surgery or radiotherapy will proceed as planned per standard care.
- If 1-5 new lesions are detected, your doctor will adjust your treatment to include the new lesions in the treatment plan. In this case, additional visits may be needed to complete the treatment.
- If more than 5 lesions are detected, your doctor may change or simply cancel your surgery or radiotherapy, so that treatment can be focus on more effective treatment in this circumstance.

<u>- Groups 1 and 2 (all participants)</u>

You will undergo surgery or radiotherapy (with or without hormonal therapy). The number of visits will vary based on the specific treatment planned. You doctor will let you know how many visits your treatment will entail.

3) Follow-up phase (7 visits, 4 hours each)

At 3 and 6 months after your treatment, and annually thereafter for a total of 5-8 years, the following procedures will be performed **for both study groups**.

- A blood test (5mL, or 1 teaspoon) to measure your PSA if a recent measurement is not available.
- A targeted medical examination, as needed, based on your medical history and current symptoms.
- Three (3) surveys to complete by internet regarding your general wellbeing, as well as your urinary, intestinal, sexual, and hormonal symptoms at 3 and 6 months, and 1,3 and 5 years after treatment. Each survey will take approximately 5-10 minutes to complete. You may find some questions uncomfortable to answer and you can ignore all questions that you do not wish to answer (total time 20-30 minute).

YOUR RESPONSIBILITIES AND IMPORTANT PRECAUTIONS TO BE TAKEN

In signing this consent form, you accept to follow the instructions of your study doctor, to undergo the study visits, and to proceed with any evaluation needed as a part of this study.

- You must inform your study doctor or a member of the team as soon as possible if you develop unusual symptoms or side effects, as this may have an impact on your health. You can reach then at the telephone number indicated in section «Contact Resources».
- In case of emergency (evening, night, weekends, holidays) in order to report side effects linked to the study, you must present yourself to the emergency room as needed and you will be seen by the doctor on call in radiation-oncology or urology. You must mention that you are participating in a research study.

RISKS AND INCONVENIENCES RELATED TO PROCEDURES

Groups 1 and 2 (all participants)

- **Risks linked to surgery (if planned in your case):**
  - **More frequent (10% or more)**

- Temporary catheter and drain
- Erectile dysfunction after injury to nerves
- Infertility

  - **Less Frequent (1-10%)**

- Blood loss requiring transfusion or second surgery
- Temporary or permanent urinary incontinence requiring a prosthesis or other intervention.

  - **Rare but serious (less than 1%)**

- Problems related to anaesthesia or cardiovascular problems leasing to hospitalisation in intensive care (such as pneumonia, pulmonary embolism, stroke, deep vein thrombosis, heart attack)
- Pain, injection or hernia near the incision.
- Rectal injury requiring very rarely a colostomy (temporary)

- **Risks linked to radiotherapy (if planned in your case):**

  - **More frequent (10% or more)**

- Urinary frequency or urgency (temporary)
- Discomfort or burning in urinating (temporary)
- Increased frequency and/or change in stool consistency (temporary)
- Light fatigue
- Erectile dysfunction

  - **Less frequent (1-9%):**

- Incontinence of stool
- Rectal bleeding (normally light)
- Chronic bladder or bowel symptoms as described above
- Temporary urinary retention requiring use of catheter

  - **Rare but serious (1% or less)**

- Injury to the bladder or rectum requiring surgery

-Risks linked to hormonal therapy (if planned in your case)

  - **More frequent (10% or more)**

- Hot flashes
- Erectile dysfunction
- Loss of libido
- Light fatigue
- Light sensitivity or swelling of breasts
- Diarrhea
- Reduction in bone density (note, patients receving long term hormonal therapy (32 months) may be at higher risk)

  - **Less frequent (1-10%)**

- Headache
- Bone or joint pain
- Liver toxicity (found in a blood test) requiring a reduction in dose or discontinuation of hormonal therapy

Group 1 only – PSMA-PET

- **Risks related to the PSMA-PET scan**

Radiotracers, including PSMA, generally to not cause side effects. Allergic reaction are excessively rare (rash, but a severe allergic reaction has never been described).

During the PSMA-PET scan, you will be exposed to a small dose of radiation. The estimated dose you will receive during the test is 10-12 mSv (dose may vary depending on your weight, which influences the parameters applied during the test). Individually, this exposure has no significant risk of side effects. However, repeated exposures to radiation may cause side effects (higher risk of cancer linked to exposure to radiation). People are exposed daily to a dose of radiation in the environment (roughly 1,6 mSv per year in Montreal). To compare, a plane trip from Montreal to

Paris exposes a person to 0.06 mSv, a chest x-ray 0.1 mSv and some scans 15 mSv.

- **Risks and discomforts with intravenous injection**

-More frequent (10% or more)

You may feel discomfort during intravenous injection:

- Sharp pain during needle insertion
- Slight pressure or pulling

-Less frequent (1%)

Possible side effects related to intravenous injection my include

- Bruises
- Bleeding
- Extravasation (spread of the medicine in the tissue around your vein)

- **Risks and discomforts liked to Lasix (medicine to help urinate)**

-More frequent (more than 10%)

- Dehydration
- Drop in blood pressure
- Dizziness

-Frequent (between 1 and 10%)

- Electrolyte imbalance in your blood

-Less frequent (less than 1%)

- Nausea, vomiting, painful urinary retention, kidney failure, or allergic reaction.

Risks and side effect liked to adjustments in your surgery or radiotherapy will depend on the region of your body where the treatment is targeted. Your study doctor will discuss in detail with you these possible side effects. Examples of the more common side effects, based on the region in your body) are listed below:

-Risks linked to additional radiotherapy to bone (if needed)

- **More frequent (more than 10%)**

- Skin irritation

- **Frequent (between 1 and 10%)**

- Pain

- **Less frequent (less than 1%**

- Weakening of the bone leading to fracture
- Loss of hair may be permanent in the section of skin exposed
- Redness, rash, or desquamation of skin

-Risks linked to additional radiotherapy to the spine (if needed)

- **More frequent (more than 10%)**

- Temporary flare of bone pain
- Nausea

- **Frequent (between 1 and 10%)**

- Inflammation of oral mucosa and esophagus (tube between your mouth and stomach), leading to trouble swallowing, and if you cannot swallow water, dehydration my occur needing intravenous rehydration.
- Inflammation of your breathing tube including your vocal cords, which may cause a hoarse voice or a loss of your voice.
- Inflammation of your lungs, which can lead to cough, thick mucus, difficulty breathing, or pneumonia
- Fracture or compression of vertebra, which can cause pain and a need for surgical or non-surgical treatment.

- **Less frequent (less than 1%)**

- Esophageal fistula (abnormal opening in the tube between your mouth and your stomach).
- Scarring of the gut, which can lead to blockage needing surgery.
- Temporary or permanent injury to the spinal cord, which may lead to abnormal sensation in the skin such as burning, tingling, or muscle weakness cause inability to walk (paralysis)

- **Risks linked to additional radiotherapy to the abdomen or pelvis (if needed)**

- **More frequent (more than 10%)**

- Fatigue (which resolved avec radiotherapy is completed)
- Nausea

- **Frequent (between 1 and 10%)**

- Irritation of the skin (redness, ulcers, itching and discomfort)
- Irritation of the gut (bleeding, blockage, fistula(abnormal connection with other tissues), or change in bowel habit, like diarrhea, needing treatment with medications or surgery)
- Kidney damage which may require medical treatment.

-Risks linked to a more extensive surgery (if needed)

- Frequent (1-10%)

- Bleeding requiring a transfusion, embolization or second surgery
- Lymphocele which may require drainage
- Deep vein thrombosis which my require anticoagulation

-Less frequent (less than 1%)

- Swelling in your legs needing compression stockings, lymphatic massage, or leg raise.
- Injury to the obturator nerve needing surgical repair, or physiotherapy after surgery
- Injury to the bowel needing surgical repair, drainage, or a prolonged hospitalisation
- Injury to ureters needing surgical repair, nephrectomy, nephrostomy, or prolonged drainage.

RISKS RELATED TO SEXUAL ACTIVITY AND PROCREATION

You should avoid close contact with other, including sexual activity, for the 12 hours following the PSMA-PET. If your partner becomes pregnant after the PSMA-PET SCAN, you will have to inform your study doctor. Since the risk to your partner and baby are unknown, it is advisable that your partner accepts medical follow-up for her during pregnancy and for the baby after birth. If you agree, you should ask your partner to sign a consent form authorizing the doctor to forward to the study doctor the data on her health during pregnancy and the baby's health at birth

BENEFITS

You may obtain a personal benefit from your participation in this study but we cannot guarantee it. At the very least, the results obtained will contribute to the progress of the knowledge in this field.

CONFIDENTIALITY

During your participation in this study, the study doctor and his team will collect and record information about you in a study file. They will only collect information required to meet the scientific goals of the study

The study file may include information from your medical chart concerning your past and current state of health, your lifestyle, as well as the results of the tests, exams, and procedures that you will undergo during this research project. Your research file could also contain other information, such as your name, sex, date of birth and ethnic origin.

All the information collected during the research project will remain strictly confidential to the extent provided by law. You will only be identified by a code number. The key to the code linking your name to your study file will be kept by the principal investigator.

To ensure your safety, a copy of this information and consent form will be placed in your medical chart. As a result, any person or company whom you give access to your medical chart will have access to this information.

The researcher responsible for this research project will send to Lantheus Inc. collaborator anonymized images (that is to say that directly and indirectly identifying information will be suppressed so as to make it impossible in principle to identify people) taken during the research PET-PSMA exam. No other data about you will be shared.

The collaborator will respect the confidentiality rules in effect in Quebec and Canada, regardless of the country.

The study data will be stored for at least 10 years by the researcher responsible for this research project

The study data may be published or shared during scientific discussions; however it will not be possible to identify you.

For monitoring, control, safety, security, and marketing of a new study drug, your study file as well as your medical charts may be examined by a person mandated by Canadian or international regulatory authorities, such as Health Canada, as well as by representatives of the sponsor, the institution, or the Research Ethics Board. All these individuals and organizations adhere to policies on confidentiality.

You have the right to consult your study file in order to verify the information gathered, and to have it corrected if necessary.

COMMUNICATION OF OVERALL RESULTS

You can find out the overall results of this study if you ask the principal investigator at the end of the study.

COMPENSATION

You will not receive financial compensation for participating in this research study and this participation could cause you additional costs (parking, gas, meals and taxis).

SHOULD YOU SUFFER ANY HARM

Should you suffer harm of any kind following administration of the study drug, or following any other procedure related to the research study, you will receive the appropriate care and services required by your state of health

By agreeing to take part in this study, you are not giving up any of your rights, nor are you releasing the researcher responsible for the research project, sponsor or establishment where the study is being conducted from their civil and professional liability.

VOLUNTARY PARTICIPATION AND THE RIGHT TO WITHDRAWAL

Your participation in this research project is voluntary. Therefore, you may refuse to participate. You may also withdraw from the project at any time, without giving any reason, by the research team.

Your decision not to participate in the study, or to withdraw from it, will have no impact on the quality of care and services to which you are otherwise entitled, or on your relationship with the clinical team providing them.

The study doctor or the Research Ethics Board may put an end to your participation without your consent. This may happen if new findings or information indicate that participation is no longer in your interest, if you do not follow study instructions, or if there are administrative reasons to terminate the project.

However, before you withdraw from the study or are withdrawn from the study, the information collected during the study will nonetheless be stored, analyzed or used to protect the scientific integrity of the research project.

If you withdraw from the study or are withdrawn from the study, the information collected during the study will nonetheless be stored, analyzed or used to protect the scientific integrity of the research project.

Any new findings that could influence your decision to stay in the research project will be shared with you as soon as possible.

ALTERNATIVE TREATMENTS

If you do not wish to take part in the study, your treating doctor will discuss your treatment options with you.

IDENTIFICATION OF CONTACT RESOURCES

If you have questions, or experiencing problems related to the research project, or if you wish to withdraw, you can contact Dr. Cynthia Ménard, responsible researcher from Monday to Friday between 8h00 and 16h00 at (514) 890-8254.

For any question concerning your rights as a research subject participating in this research project or if you have comments or wish to file a complaint, you can contact the CHUM commissioner for complaints and service quality at 514-890-8484.

INFORMATION RELATED TO THE STUDY ON THE INTERNET

A description of this study is available in English only on the web site http://www.ClinicalTrials.gov. This site does not contain any information that could that could identify you. The site will mainly include a summary of the results when they become available. You can check this web site any time. The registration number for this project is NCT04557501.

SIGNATURE

I have reviewed the information and consent form. Both the research study and the information and consent form were explained to me. My questions were answered, and I was given sufficient time to make a decision. After reflection, I consent to participate in this research study in accordance with the conditions stated above.

I authorize the research study team to have access to my medical record for the purposes of this study.

Name (Please print)	Signature of the participant	Date

SIGNATURE OF THE PERSON WHO OBTAINED THE CONSENT

I have explained to the research subject the terms of the nature and purpose of this study and the risked involved, and answered the questions asked.

Name (Please print)	Signature of the person obtaining consent	Date

COMMITMENT OF THE PRINCIPAL INVESTIGATOR

I certify that this information and consent form was explained to the research participant, and that the questions the participant had were answered.

I undertake, together with the research team, to respect what was agreed upon in the information and consent form, and to give a signed and dated copy of this form to the research participant

WITNESS SIGNATURE

YES □ NO □

Signature of a witness is required for the following reasons:

- □ Difficulty or inability to read - The person (independent witness) who places it signature below certify that they have read the consent form and explained exactly the project to the participant, who seems to have understood.
- □ Misunderstanding of the language of the consent form - the person who signs below acted as interpreter for the participant in the process of obtaining consent.

Name (printed letters)	Witness signature	Date

Please note:

Any additional information about assistance given to the participant during the consent process must be noted in his/her research file.

APPROVAL OF THE RESEARCH ETHICS BOARD

The CHUM Research Ethics Board approved this study and is responsible for the monitoring it for centers participating within Quebec.

Tests and Procedures

	**Before treatment**	**During treatment**	**Follow-up**						
Tests and procedures			3 months	6 months	1 year	2 years	3 and 4 years	5 years	6-7-8 years
Survey	x		x	x	x	x		x	
PSMA-PET (group 1)	x								
Blood test	x		x	x	x	x	x	x	x
Symptom Evaluation	x	x	x	x	x	x	x	x	x
